# Targeted Next-Generation Sequencing in Patients with Suggestive X-Linked Intellectual Disability

**DOI:** 10.3390/genes11010051

**Published:** 2020-01-02

**Authors:** Nekane Ibarluzea, Ana Belén de la Hoz, Olatz Villate, Isabel Llano, Intzane Ocio, Itxaso Martí, Miriam Guitart, Elisabeth Gabau, Fernando Andrade, Blanca Gener, María-Isabel Tejada

**Affiliations:** 1 Biocruces Bizkaia Health Research Institute, 48903 Barakaldo, Spain; 2 Spanish Consortium for Research on Rare Diseases (CIBERER), 28029 Madrid, Spain; 3 Genetics Service, Cruces University Hospital, Osakidetza Basque Health Service, 48903 Barakaldo, Spain; 4 Department of Paediatric Neurology, Araba University Hospital, Osakidetza Basque Health Service, 01009 Gasteiz, Spain; 5 Department of Paediatric Neurology, Donostia University Hospital, Osakidetza Basque Health Service, 20014 Donostia, Spain; 6 Genetics Laboratory, Paediatric Unit, Parc Taulí Hospital Universitari, Institut d’Investigació i Innovació Parc Taulí I3PT, Universitat Autònoma de Barcelona, 08208 Sabadell, Spain

**Keywords:** X-linked intellectual disability, next-generation sequencing, gene panel, *HUWE1*, *IQSEC2*, *MED12*, *PHF8*, *SLC6A8*, *SLC9A6*, *SYN1*

## Abstract

X-linked intellectual disability (XLID) is known to contribute up to 10% of intellectual disability (ID) in males and could explain the increased ratio of affected males observed in patients with ID. Over the past decade, next-generation sequencing has clearly stimulated the gene discovery process and has become part of the diagnostic procedure. We have performed targeted next-generation sequencing of 82 XLID genes on 61 non-related male patients with suggestive non-syndromic XLID. These patients were initially referred to the molecular genetics laboratory to exclude Fragile X Syndrome. The cohort includes 47 male patients with suggestive X-linked family history of ID meaning that they had half-brothers or maternal cousins or uncles affected; and 14 male patients with ID and affected brothers whose mothers show skewed X-inactivation. Sequencing data analysis identified 17 candidate variants in 16 patients. Seven families could be re-contacted and variant segregation analysis of the respective eight candidate variants was performed: *HUWE1*, *IQSEC2*, *MAOA*, *MED12*, *PHF8*, *SLC6A8*, *SLC9A6,* and *SYN1*. Our results show the utility of targeted next-generation sequencing in unravelling the genetic origin of XLID, especially in retrospective cases. Variant segregation and additional studies like RNA sequencing and biochemical assays also helped in re-evaluating and further classifying the genetic variants found.

## 1. Introduction

Intellectual disability (ID) is the most common neurodevelopmental disorder with a worldwide prevalence of about 1% [1,2] and is defined by impaired cognitive functioning and adaptive behavior arising before the age of 18 [3]. Its severity is usually measured by the intelligence quotient (IQ) score and classified as mild (IQ 55–70), moderate (IQ 40–55), severe (IQ 25–40), or profound (IQ < 25). Clinically, it has also been useful to divide into two other categories: Syndromic and non-syndromic or unspecific intellectual disability [4]. Syndromic forms of ID show dysmorphic, neurological, or systemic features that are recognizable by medical geneticists. Conversely, non-syndromic or unspecific forms only show ID as a common feature although they may individually show additional clinical features. Yet, the distinction between the two categories is obscure and sometimes arbitrary [5,6,7].

X-linked intellectual disability (XLID) has captured great attention due to the overrepresentation of males in intellectual disability [1]. Scientists have spent a good deal of energy in deciphering the genetic origin of XLID by applying different techniques. Linkage analysis followed by candidate gene testing has been a popular technique in screening large families with X-linked inheritance of ID [7,8]. Over the past decade, sequencing studies have clearly stimulated the gene discovery process [9]. Currently, roughly 141 genes are known to be associated with XLID [10]. Regardless, the aberrant expansion of the CGG trinucleotide repeat at the *FMR1* gene that causes Fragile X Syndrome (FXS) is still the most common condition in XLID [11].

Owing to the genetic heterogeneity of XLID, next-generation sequencing technologies have become of great benefit in this field. Tarpey et al. (2009) [12] set the grounds for next-generation sequencing in XLID by sequencing all coding exons of the X-chromosome and reaching a diagnostic yield of 25%. Both XLID gene panel [13] or X-exome sequencing strategies [14,15,16] have been applied since then in families with suggestive X-linked inheritance of ID [12,14,16] and affected male sib pairs or sporadic cases [13,15] obtaining a similar diagnostic yield. However, many of the XLID families tested through X-exome or gene panels remained without diagnosis. Whole-exome sequencing of patients with suggestive XLID has demonstrated that ID could be of autosomal origin in these patients and occur de novo despite family history [17]. Indeed, exome sequencing has been demonstrated to increase the diagnostic yield in unresolved patients with ID, highlighting the importance of de novo mutations [9,18]. Despite the popularity of whole-exome sequencing in intellectual disability, gene panels are still useful in clinical practice.

In this study, we have targeted 82 XLID genes by next-generation sequencing in a cohort of 61 patients with suggestive non-syndromic XLID to try to elucidate the genetic origin of ID.

## 2. Materials and Methods

### 2.1. Patient Cohort

Patients’ samples have been received since 1992 in our molecular genetics laboratory for Fragile X Syndrome testing from pediatric neurology units, neurologists, or medical geneticists, mostly of the 4 public hospitals of the Basque Autonomous Community (Spain) because they had been diagnosed with global developmental delay or unspecific intellectual disability. Clinical data for *FMR1* testing were generally collected using a standardized questionnaire that briefly included information on clinical and family history and phenotype. Written informed consent was obtained from patients’ parents or legal representatives prior to genetic testing and this study was approved by the Research Ethics Committee at Cruces University Hospital (CEIC: E18/07).

For the purpose of this study, 47 unresolved male patients with possible non-syndromic XLID were selected based on the X-linked family history of ID, meaning that they had affected half-brother and/or uncle/nephew and/or maternal male cousin. Furthermore, 14 index males with ID and affected brothers were also selected based on the mother’s skewed X-inactivation pattern. All 61 patients had normal G-banded karyotype and showed normal *FMR1* trinucleotide repeat numbers. Additional molecular techniques have also been applied in some of them (Table 1).

According to the clinical data collected for the *FMR1* test, the probands were aged 2–63 in the XLID group and 2–24 in the male siblings group, 55.32% and 64.29% of the patients being under 10 at the time of referral in both groups, respectively. IQ evaluation had not been performed in 42.55% and 50% of the patients or was unknown, and those that already had an IQ evaluation mainly showed mild to borderline intellectual disability (34.04% and 35.71%, respectively). Most of the selected patients did not have relevant dysmorphic features, confirming that they show an unspecific phenotype. However, autism spectrum disorders were present in nearly 30% of them. Almost 15% of the patients in the X-linked group also reported having epilepsy. Table 1 summarizes all of these data.

### 2.2. X Inactivation

X chromosome inactivation on peripheral blood was assessed on the available samples of the selected patients’ mothers by genotyping the highly polymorphic small tandem repeat within the 5′ UTR of the human androgen receptor (AR) gene following the protocol described by Allen et al. (1992) [19]. Inactivation was considered to be random if the ratio of active to inactive X was less than 75:25. Extreme skewing of X inactivation was defined as the preferential inactivation of one X chromosome in 90–95% of cells [20].

### 2.3. Targeted Next-Generation Sequencing

To create a cost-efficient gene panel that could be transferable to XLID diagnosis in the clinic, we selected 82 X-linked intellectual disability genes based on the available literature at the moment. The selected gene panel includes genes that can lead to either non-syndromic XLID or high phenotypic variability in males and candidate genes that have been reported in XLID but have not yet been established as XLID genes (see Table S1). Genes leading to syndromic forms that show identifiable clinical features were excluded since they are generally identifiable by medical geneticists. The selected genes are the following: *ACSL4*, *AFF2*, *AGTR2*, *ALG13*, *AP1S2*, *ARHGEF9*, *ARX*, *ATP6AP2*, *ATP7A*, *ATRX*, *BRWD3*, *CASK*, *CCDC22*, *CLIC2*, *CUL4B*, *DCX*, *DLG3*, *DMD*, *FAM120C*, *FLNA*, *FMR1*, *FTSJ1*, *GDI1*, *GPC3*, *GPC4*, *GRIA3*, *HCFC1*, *HDAC8*, *HSD17B10*, *HUWE1*, *IGBP1*, *IL1RAPL1*, *IQSEC2*, *KAL1*, *KDM5C*, *KDM6A*, *KIAA2022*, *L1CAM*, *MAOA*, *MECP2*, *MED12*, *MID1*, *NAA10*, *NDP*, *NHS*, *NLGN3*, *NLGN4X*, *NSDHL*, *OFD1*, *OPHN1*, *PAK3*, *PHF6*, *PHF8*, *PLP1*, *PQBP1*, *PRPS1*, *PTCHD1*, *RAB39B*, *RBM10*, *RBMX*, *RNF113A*, *RPL10*, *RPS6KA3*, *SLC16A2*, *SLC6A8*, *SLC9A6*, *SMARCA1*, *SMC1A*, *SMS*, *SRPX2*, *SYN1*, *SYP*, *TAF1*, *TSPAN7*, *UBE2A*, *UPF3B*, *USP9X*, *WDR45*, *ZC4H2*, *ZDHHC15*, *ZDHHC9*, *ZNF711*.

Genomic DNA target enrichment was performed by PCR based amplification using the custom X-linked Intellectual Disability gene panel. Ion Ampliseq^TM^ Designer (Life Technologies, Foster City, CA, USA) was used to design primer pairs to amplify all exons and flanking regions of the selected 82 genes. The designed gene panel generates 2471 amplicons of 125–375 bp resulting in 98.71% in silico coverage of all the regions of interest. DNA library was prepared by multiplex PCR, using the Ion Ampliseq^TM^ Library kit v2.0 kit (Life Technologies, Foster City, CA, USA). Sequencing templates were built using Ion One Touch^TM^ 2 System (Life Technologies, Foster City, CA, USA) and then sequenced using the Ion PGM^TM^ System (Life Technologies, Foster City, CA, USA) with Hi-Q View chemistry. Torrent Suite^TM^ Software (Life Technologies, Foster City, CA, USA) was used for sequencing raw data analysis, alignment to the human reference genome (hg19/GRCh37), and variant calling (Torrent Variant Caller plug-in). Variants were also called, annotated, filtered, and analyzed on the Ion Reporter^TM^ Software version 5.2 (Life Technologies, Foster City, CA, USA).

Variants below 10× coverage and 30 Phred score were excluded due to quality reasons. Furthermore, common variants and variants with a >0.005 minor allele frequency (MAF) were filtered out. Variants that appeared more than 2 times in our cohort were also excluded. Finally, non-synonymous variants in exonic and splice site regions were also prioritized. In order to predict the pathogenicity of the genetic variants found, different in silico predictors such as SIFT [21], Polyphen-2 [22], and MutationTaster-2 [23] were used. CADD scores [24] were also calculated for every variant. Variants in splice-site regions were evaluated using in silico predictors like Human Splicing Finder [25] and NNSplice [26].

Candidate variants were then validated by conventional Sanger sequencing (Primer sequences available on request) and were then classified following the American College of Medical Genetics (ACMG) guidelines [27] and using the InterVar software [28]. 

Novel sequence variants have been deposited in GenBank. Accession numbers are provided on the results section.

### 2.4. Variant Segregation and Additional Analysis

Variant segregation of the identified candidate variants was performed when possible. For this purpose, families were re-contacted. Variants were confirmed by conventional Sanger sequencing, as previously described.

Putative splicing mutations were also tested using blood RNA. Briefly, RNA was extracted from peripheral blood and cDNA was obtained using Superscript RT II enzyme (Invitrogen, Carlsbad, CA, USA) from 1 µg of total RNA extracted in a volume of 20 µL. cDNA was then amplified and sequenced to identify potential splicing variants.

Additional biochemical studies were also performed when required. In this way, plasma creatine, creatinine, and guanidoacetate levels were measured by Liquid chromatography—tandem mass spectrometry (LC-MS/MS) following the protocol described by Bodamer et al. (2001) [29] to determine creatine deficiency syndrome.

## 3. Results

### 3.1. Targeted Next-Generation Sequencing

Targeted sequencing generated a mean of 788.702 reads per sample with a 255 bp mean read length, which covered 96.31% of the target regions with a 281.46 mean read depth, and 97% of the sequenced regions were covered at least 20× (Detailed data on technical sequencing is included on Table S2). Not covered regions mainly include GC-rich regions like 3′ or 5′ UTR regions, which are already known to be a burden in next-generation sequencing.

A mean of 188 variants per patient were annotated. Variants were then filtered and prioritized as described before. In total, 17 variants were prioritized as candidate (Table 2), 12 of them were identified in 11 patients that showed suggestive X-linked inheritance and 5 in male sib-pairs. These include 14 missense variants, 1 nonsense, 1 splice-site variant, and an in-frame deletion. All of the variants had been inherited from the mother and most of them were not annotated in any of the population databases like GnomAD, although others were. Indeed, missense variants in *UPF3B* (c.1118G>A; p.Arg373His); *DLG3* (c.1424C>T; p.Ser475Leu), *FMR1* (c.1816C>T; p.Arg606Cys); *HUWE1* (c.1125G>T; p.Met375Ile) and *CCDC22* (c.1388C>G; p.Ala463Gly) have been identified in hemizygous males in GnomAD and dbSNP (Table 2) and the variant c.1118G>A in *UPF3B* has already been reported in ClinVar as variant of unknown significance (VUS). Although there is no detailed information on frequency data, variants in *MAOA* (c.617G>A; p.Arg206Gln), *PRPS1* (c.611G>A; p.Arg204His), and *SYN1* (c.796G>A; p.Val266Met) have also been reported in dbSNP (rs1218703391, rs1169615098, rs1327735600).

On the other hand, two possible truncating variants have been identified and classified as pathogenic: *PHF8* (c.252C>A; p.Tyr84*) and *UPF3B* (c.371-1G>C). An in-frame deletion in *SLC6A8* (c.1390_1392delGAT; p.Asp464del) has also been identified and classified as likely pathogenic. The other six missense variants found have been classified as VUS since they have not previously been reported on the literature and more information on the function or segregation analysis is needed. This is why we made an effort in re-contacting the families.Accession numbers of the novel sequence variants that have been analyzed in this study (Table 2) are the following: *PHF8* (c.252C>A; p.Tyr84*) MN817111, *UPF3B* (c.371-1G>C) MN817115, *SLC6A8* (c.1390_1392del GAT; p.Asp464del) MN817113, *IQSEC2* (c.128G>C; p.Arg43Pro) MN817117, *SLC9A6* (c.316A>G; p.Met106Val) MN817110, *NHS* (c.1270A>G; p.Arg424Gly) MN817112, *CASK* (c.490G>A; p.Gly164Arg) MN817116, *HUWE1* (c.12209C>G; p.Ser4070Cys) MN817114, and *MED12* (c.5009C>T; p.Ser1670Phe) MN817118.

### 3.2. Segregation Analysis and Genotype-Phenotype Correlation

Segregation analysis was performed on seven families and variants were reclassified based on the results obtained. Thanks to updating the pedigree and the clinical data of the patients, a genotype–phenotype correlation has been possible.

#### 3.2.1. Pathogenic/Likely Pathogenic Variants

##### Family ID0707

The proband is an eight-year-old male, second child of healthy non-consanguineous parents. He was first referred at four years of age to our laboratory because of developmental delay and autistic features and an older brother presenting with global developmental delay. He had no oral speech and communicated with signs, had no sphincter control, and did not tolerate solid food. He had minor dysmorphic features such as borderline low-set ears, thick and abundant hair, broad eyebrows with a medial eyebrow flare, small nose because of small nares, and no cleft palate or nasal voice. He had a history of repetitive otitis that caused unilateral hearing loss. Likewise, he had distal pectus excavatum, no lumbar lordosis or scoliosis, and normal genitalia. Array CGH results were normal.

**Figure 1:**
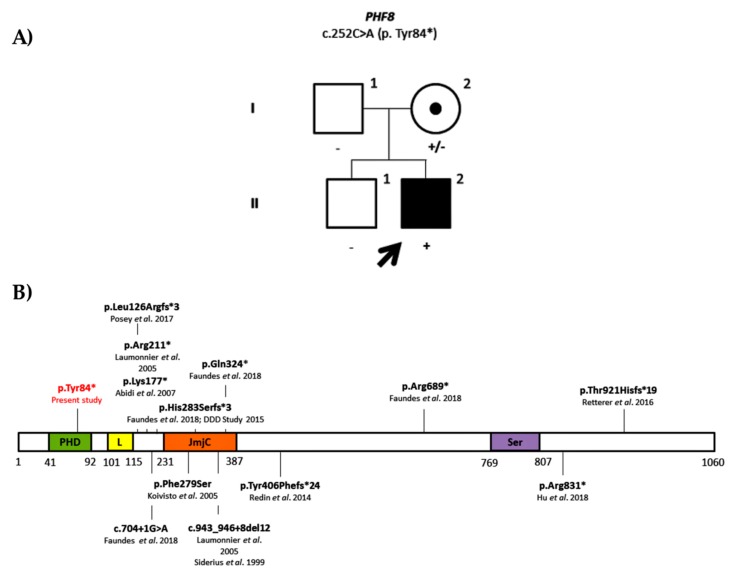
(**A**) Family pedigree of the ID0707 proband. Segregation analysis of the nonsense variant in *PHF8* gene (NM_001184896.1: c.252C>A; p. Tyr84*). (**B**) Histone lysine demethylase PHF8 protein. Protein domains are shown in color: In green, plant homeodomain (PHD) finger domain; in yellow, linker region (L); in orange, jumonji-C (JmjC) domain; and in purple, serine-rich domain (Ser). All the mutations identified to date are also shown with the respective references. The present variant is shown in red.

We identified a nonsense variant in the *PHF8* gene (NM_001184896.1: c.252C>A; p. Tyr84*). This variant was not present in any population databases and has neither been reported on the literature. The variant was inherited from his mother who had a skewed X-inactivation and was not present on the brother (Figure 1A). Despite the brother being reported as having global developmental delay in his early childhood, he eventually did not develop ID and currently has a normal development. There was no other information on the maternal family as she had been adopted. Mutations in *PHF8* were first described by Laumonnier et al. (2005) [30] and are known to cause Siderius syndrome (first described by Siderius et al. in 1999 [31]). Siderius syndrome (OMIM#300263) is characterized by mild to borderline intellectual disability associated with cleft lip/palate. Moreover, patients with this syndrome may also present with minor dysmorphic features including preaxial polydactyly, large hands, and cryptorchidism. In total, 12 truncating variants, either nonsense, frameshift, or splicing variants, have been reported in this gene in human gene mutation database (HGMD) (Figure 1B) [32,33,34,35,36,37,38,39]. The present proband does not present cleft lip or palate or any of the classical features observed in Siderius syndrome. Still, it is important to mention that there are just a few clinical reports on the identified *PHF8* variants apart from the initial reports that described the syndrome. Although the proband does not match the initial phenotype described, the identification of an early truncating variant on the proband suggests that it is a pathogenic variant (PVS1, PM2, PP3).

##### Family ID1122

The proband is a 19-year-old male with intellectual disability, autistic features, behavioral disturbances, and generalized epilepsy. He was first referred to our laboratory at two years of age with global developmental delay and family history of X-linked intellectual disability because he had two maternal uncles affected with severe intellectual disability and epilepsy. X-linked intellectual disability MLPA and array CGH results were normal.

**Figure 2:**
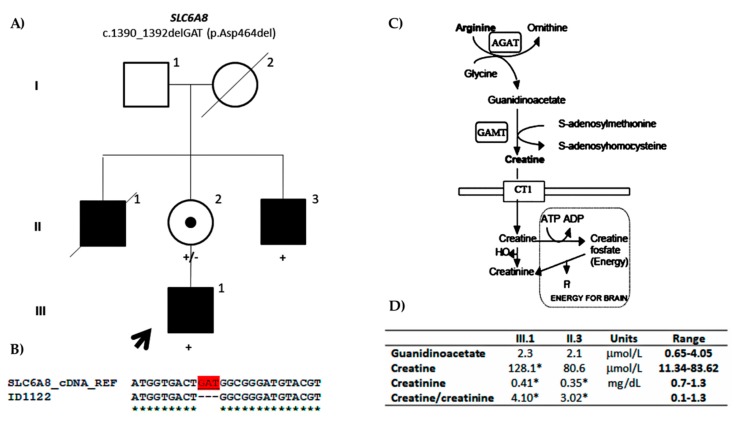
(**A**) Family pedigree of the ID1122 proband. Segregation of the c.1390_1392delGAT in-frame deletion in *SLC6A8* (NM_005629.3) is shown. (**B**) Partial cDNA sequence of the *SLC6A8* gene. cDNA sequence of the proband aligned to the reference *SLC6A8* cDNA sequence is shown. The deleted nucleotides are shown in red. (**C**) Creatine metabolism pathway (derived from Andrade et al. 2008) [42]. The protein encoded by *SLC6A8* is named creatine transporter (CT1) and it is a transmembrane protein that is responsible for the creatine uptake. (**D**) Plasma levels of guanidinoacetate, creatine, and creatinine in the proband (III.1) and affected maternal uncle (II.3) are shown together with the creatine/creatinine ratio. The asterisk indicates the values that fall out of the reference range values.

We identified an in-frame deletion in the *SLC6A8* gene (NM_005629.3: c.1390_1392delGAT; p.Asp464del) that is located to the ninth transmembrane domain of the protein. The variant is not present in any of the population databases or literature and is predicted to be pathogenic. The variant also segregated with the phenotype. Indeed, the mother and affected maternal uncle were carriers of the variant (Figure 2A). The mother showed skewed X-inactivation. Being an in-frame variant located in a splicing region, it was predicted to cause changes in splicing. Nevertheless, it was confirmed by cDNA sequencing that there were no changes in splicing (Figure 2B). Anyway, the decreased plasma creatinine levels confirmed the pathogenicity (Figure 2D). Therefore, the variant was reclassified as pathogenic (PS3, PM2, PM4, PP1, PP3). Indeed, mutations in *SLC6A8* cause cerebral creatine deficiency syndrome (CCDS1: OMIM#300352). The first patient with CCD1 and a mutation in *SLC6A8* gene was described by Salomons et al. (2001) [40] presenting with a similar phenotype as our proband, and since then, many families have been described, some of them with in-frame deletions as our family. The prevalence of *SLC6A8* mutations in males with ID has been estimated to be about 1% [41], but it could be higher. It is interesting to mention that our patients showed normal low levels of plasma creatine but not outside the normal range, so the clinicians never suspected this syndrome until the variant was found and more accurate creatine/creatinine measures were performed.

##### Family ID1208

The proband is a 30-year-old male who was referred to our laboratory at five years of age because he had global developmental delay, autistic features, and epilepsy. He also had X-linked family history of intellectual disability meaning that his half-brother and two maternal uncles of the mother were also affected with intellectual disability. Results for *MECP2, CDKL5,* and *ARX* screenings as well as MLPA for X-linked intellectual disability and subtelomeric regions were normal.

**Figure 3:**
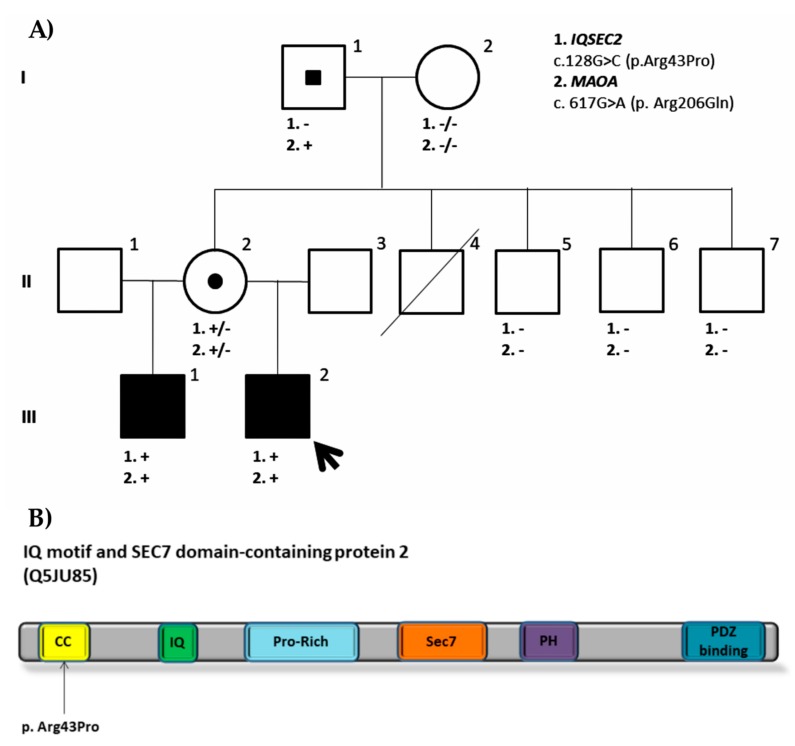
(**A**) Family pedigree of the ID1208 proband. Segregation analysis of both missense variants found in *IQSEC2* (NM_001111125.2: c.128G>C; p.Arg43Pro) and *MAOA* (NM_000240.3: c. 617G>A; p. Arg206Gln) is shown. (**B**) IQ motif and SEC7 domain-containing protein 2. Protein domains are shown in color: In yellow, coiled coil domain (CC); in green, IQ domain (IQ); in light blue, proline-rich (Pro-Rich) motif; in orange, Sec7 domain (Sec 7); in purple, Pleckstrin homology (PH) domain; and in blue, PDZ binding domain (PDZ binding). The missense variant found and located to the CC domain is also represented.

We identified two candidate variants: *IQSEC2* (NM_001111125.2) c.128G>C (p.Arg43Pro) and *MAOA* (NM_000240.3) c. 617G>A (p. Arg206Gln). While *IQSEC2* variant is a novel variant and therefore is not present in any population databases, *MAOA* variant was found in dbSNP (rs1218703391). Both variants are located in conserved amino acid residues located and are predicted to be likely pathogenic by in silico predictors. *IQSEC2* and *MAOA* missense variants are both present on the half-brother as well as on the healthy mother, who has random X inactivation. While *IQSEC2* variant occurred de novo on the mother, the *MAOA* variant had been inherited from the healthy maternal grandfather, suggesting that it is a likely benign variant (PM1, PM2, PP3, BS2, BP5). None of the healthy maternal uncles or grandmother are carriers of any of these variants (Figure 3A). The obtained results are clearly reflected on the phenotypes observed. Both the proband and his half-brother share the same phenotype: Severe to profound intellectual disability, no speech or language, autistic features, and generalized epilepsy. The proband shows minor dysmorphic features that include synophrys, everted lower lip vermilion, and kyphoscoliosis, and his brother also shows scoliosis besides unspecific dysplastic features in the facies and feet. Mutations in *IQSEC2* were first described in families with non-syndromic XLID [43] and affected males showed moderate to severe intellectual disability, seizures, autistic traits, and behavioral disturbances as our patients do. To date, more than 70 mutations have been reported in *IQSEC2* leading to a similar phenotype [44,45]. Some of these mutations are clustered in functional domains like IQ and Sec7 domains, which have been demonstrated to impair GEF activity [44,46,47]. The present variant is located to the N terminal coiled coil (CC) domain of the protein where no pathogenic missense variants have been reported so far (Figure 3B). This CC domain is thought to promote self-assembly and its disruption is likely to influence interactions with other proteins like calmodulin or PDZ containing proteins. Knockout of this domain alters its accumulation at the post synaptic density [48,49,50]. All in all, the segregation of the variant with the phenotype together with the correlation with the phenotypes observed in patients with *IQSEC2* variants suggests that this variant is likely pathogenic (PM2, PP1, PP2, PP3, PP4). However, as it is a missense variant and no other pathogenic missense variants have been reported at the CC domain, functional studies would be needed to establish its pathogenicity and understand its disease mechanism.

#### 3.2.2. Variants of Unknown Significance

##### Family ID0216

The proband is a 27-year-old male with intellectual disability and severe behavioral disturbances. He was first referred to the molecular genetics service at six years of age because he presented with global developmental delay. He is the first child of heathy non-consanguineous parents and has a younger brother and sister, both of them healthy. He has two maternal uncles affected with mild ID. Array CGH results were normal.

**Figure 4:**
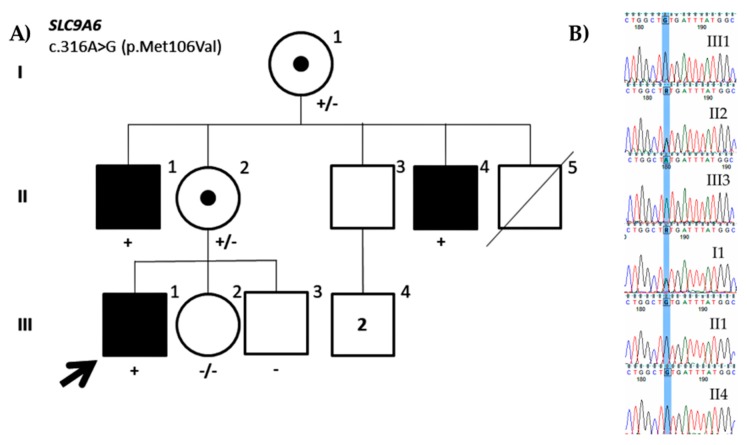
(**A**) Family pedigree of the ID0216 proband. Segregation of the missense variant identified in *SLC9A6* (NM_001042537.1: c.316A>G; p.Met106Val). (**B**) Partial DNA sequence of the SLC9A6 gene. The identified nucleotide change is highlighted.

A missense variant in the *SLC9A6* gene (NM_001042537.1: c.316A>G; p.Met106Val) was identified in this study. The variant was inherited from the mother who presented with random X-inactivation and was also present in both maternal uncles having mild ID (Figure 4). Therefore, the variant seems to segregate with the disease. Moreover, the variant has not been reported in population databases and is located on a conserved residue in the second transmembrane domain of the protein and could affect its structure and, consequently, function. Nevertheless, the in silico variant predictors do not consistently agree on its pathogenicity. Mutations in *SLC9A6* have been associated with Christianson syndrome (OMIM#300243) [51], which is mainly characterized by severe intellectual disability, no speech, postnatal microcephaly, early onset seizures, ataxia, and hyperactivity [52]. To date, more than 50 causative variants that mainly include nonsense, frameshift, splicing, and indels have been reported in HGMD. However, patients showing mild ID have also been reported [53], as it is the case of the present patient. Altogether, although the segregation of the variants suggests that the variant might be pathogenic, it would still be a VUS according to the ACMG classification criteria (PM1, PM2, PP1, BP1) until more information is available or functional assays are performed like others that have already been performed in *SLC9A6* [54,55].

##### Family ID1128

The proband is a 33-year-old male with moderate to severe intellectual disability, no speech, and behavioral disturbances including aggressive behavior who was first referred to the molecular genetics laboratory at three years of age. He has an affected monozygotic twin who has mild intellectual disability and aggressive behavior, and a healthy brother and sister. Their healthy parents are consanguineous (first cousins). A cousin of the parents, who died at 18 years of age, was also reported to have severe intellectual disability and seizures (Figure 4). Molecular studies including X-linked intellectual disability MLPA and array CGH results were normal. As parents are consanguineous, whole-exome sequencing was also performed to exclude pathogenic homozygous variants.

**Figure 5:**
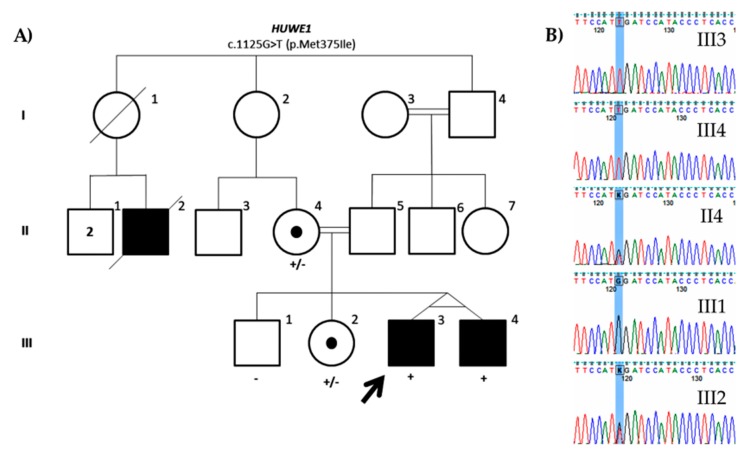
(**A**) Family pedigree of the ID1128 proband. Segregation of the missense variant found in *HUWE1* (NM_031407.6: c.1125G>T; p.Met375Ile). (**B**) Partial DNA sequence of the *HUWE1* gene. The identified nucleotide change is highlighted.

The only candidate variant identified either in the exome or gene panel is a missense variant in *HUWE1* gene (NM_031407.6: c.1125G>T; p.Met375Ile). The variant has already been reported in population databases like GnomAD with very low frequency (0.000008010) and two other variants have also been reported at the same protein residue also with a low frequency, suggesting that it could be a rare benign variant. Although it is in a conserved residue, in silico predictors suggest that it might be tolerated. As expected, the affected monozygotic twin is also carrier of the variant, as well as the mother—who shows skewed X-inactivation—and his healthy sister. The normal brother does not carry the variant (Figure 5). No more segregation analysis was performed due to the lack of collaboration of the family. *HUWE1* missense variants were first identified in patients with moderate to profound non-syndromic ID [56]. Since then, missense variants mainly clustering to the HECT domain have been reported in patients with ID. These mutations have been shown to alter expression of HUWE1 protein and its downstream targets [57,58]. Although mutations in this gene cause a wide phenotypic spectrum [59], a syndromic form has been described. A recurrent mutation in the HECT domain was associated with Juberg Marsidi and Brooks syndrome (OMIM#309590) [58,60,61], which is characterized by intellectual disability, poor or absent speech, short stature, and microcephaly. Dysmorphic features include deep-set eyes, prominent nose, and blepharophimosis. Despite *HUWE1* being highly intolerant to missense variants (*Z* = 8.87), the present variant is not located in any of the functional domains described. The variable phenotype observed in patients with *HUWE1* variants does not help in determining its pathogenicity neither. Therefore, although exome sequencing was performed and no other candidate variant was found, this variant would still be a VUS (PM1, PM2) since more evidence like a broader segregation analysis or functional assays are needed to claim its pathogenicity.

##### Family ID1402

The proband is a 21-year-old male who was referred to our laboratory at five years of age with mild intellectual disability and autistic features. He was born to healthy non-consanguineous parents and has two younger sisters. He has a maternal uncle who had school and language delay in childhood and now suffers from paranoid schizophrenia.

**Figure 6:**
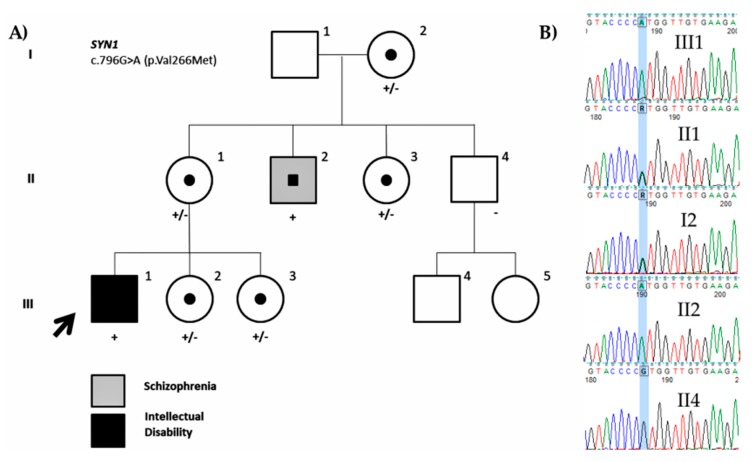
Family pedigree of the ID1402 proband. (**A**) Segregation of the missense variant in *SYN1* (NM_006950.3: c.796G>A; p.Val266Met) is shown. The black color indicates intellectual disability and the grey indicates schizophrenia. (**B**) Partial DNA sequence of the *SYN1* gene. The identified nucleotide change is highlighted.

We identified a missense variant in *SYN1* (NM_006950.3: c.796G>A; p.Val266Met). The variant was inherited from the mother and was also present on the maternal uncle affected with schizophrenia, but not on the healthy maternal uncle. The two younger sisters and maternal aunt were also carriers of the variant (Figure 6). Although this variant has already been reported (rs1327735600), no hemizygous variants have been identified. The variant is located on a conserved amino acid residue at the actin and synaptic vesicle binding region of the protein and is predicted to be deleterious. Variants in *SYN1* have been associated with learning difficulties, epilepsy, and aggressive behavior [62]. Fassio et al. (2011) [63] identified more variants both truncating and missense associated with ASD and/or epilepsy. The absence of any other members affected with ID in the family makes it difficult to assess the pathogenicity of the variant as the carrier uncle seems to show a different phenotype (low but normal IQ and schizophrenia) (Figure 6). Therefore, it should be still considered a VUS (PM1, PM2, PP3) until functional studies are performed or more information on the variant is obtained.

##### Family ID1405

The proband is a 42-year-old male referred to our laboratory for X-linked intellectual disability testing. He presents with intellectual disability, autistic features, schizophrenia, and behavioral disturbances. He has a nephew with global developmental delay and autistic features and no other family history of intellectual disability. Array CGH results were normal.

**Figure 7:**
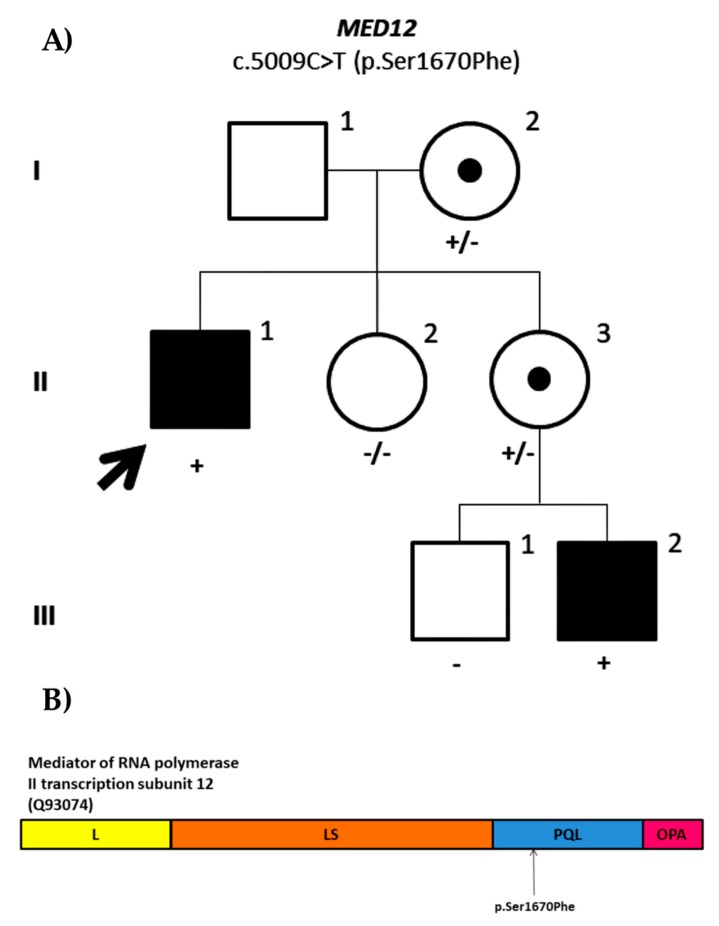
(**A**) Family pedigree of the ID1405 proband. Segregation analysis of the missense variant in *MED12* (NM_05120.2: c.5009C>T; p.Ser1670Phe). (**B**) Mediator of RNA polymerase II transcription subunit 12. The subunits of the protein encoded by *MED12* include leucine-rich domain (L) in yellow, leucine serine-rich domain (LS) in orange, proline glutamine and leucine-rich domain (PQL) in blue, and glutamine-rich domain (OPA) in pink. The variant identified in ID1405 is located on the PQL domain as shown in the figure.

We identified a missense variant in *MED12* (NM_005120.2: c.5009C>T; p.Ser1670Phe) located on the PQL domain of the protein. The variant was inherited from the mother and was also present on the affected nephew whose mother was also a carrier (Figure 7). It is a novel variant as it has not been reported in population databases or literature and is predicted to be likely pathogenic. Germline mutations in the *MED12* gene have been associated with Opitz–Kaveggia syndrome, also known as FG syndrome 1 (OMIM#305450) [64], Lujan–Fryns syndrome (OMIM#309520) [65], which overlaps with FG syndrome 1, and X-linked Ohdo syndrome (OMIM#300895) [66]. However, the more genetic variants identified, the broader is the phenotypic spectrum observed in patients with *MED12* variants, making it difficult to fit any of the syndromes described to date in the majority of cases. Therefore, it has been suggested to define it as *MED12*-related disorders rather than attributing a syndrome [67]. To date, 28 variants have been identified in HGMD, of which most of them are missense variants. The patient ID1405 shares many of the clinical features reported in *MED12*-related disorders and the segregation of the variant with ID supports the pathogenicity. Nevertheless, according to the ACMG classification criteria, this variant is still a VUS (PM2, PP1, PP3) until some functional assay is performed to assess MED12 function.

## 4. Discussion

In this study, we have analyzed a cohort of 61 patients with suggestive non-syndromic XLID through next-generation sequencing using a gene panel of 82 XLID genes. We have identified 17 variants in 16 of the probands analyzed (Table 2) and we have been able to perform segregation analysis of eight variants in seven families.

The cohort had been selected specifically for XLID, as there was a suspicion of X-linked family history of ID in 47 of the probands. Candidate variants were found in 11 probands out of 47 (Table 2) and therefore, the contribution of the X chromosome has been 23% (11/47) in these patients. Tzschach et al. (2015) [13] followed a similar approach and sequenced 107 XLID genes in a cohort of 150 male patients, that included 50 patients with suggestive XLID (affected brother or male maternal relatives) and sporadic male patients obtaining a diagnostic yield of 26% in the XLID group. Our cohort also included 14 male patients with ID with affected brothers that were selected based on their mothers’ skewed X inactivation. Five candidate variants were found (5/14 = 35%) (Table 2). Overall, we have identified candidate variants in 26% (16/61) of our cohort, similar to other targeted sequencing studies in XLID [12,13,14,16].

More candidate variants have been identified in index males with affected brothers (35%) than in patients with suggestive X-linked inheritance of ID (23%) probably due to the fact that they were selected based on skewed X-inactivation. Similar to our study, Giorgio et al. (2016) [68] selected eight males with suggestive XLID based on their mothers’ skewed X-inactivation (>80%) and performed whole-exome sequencing identifying XLID variants in 50% of the patients. In the same line, in our cohort of 61 patients, mothers of 21 showed >80% of skewing of X-inactivation (12 on the male siblings group and 9 on the X-linked group) and candidate variants were found in 7 patients (33%). X-inactivation is a gene dosage compensatory mechanism that occurs randomly at early embryonic stage in females in most of the X-linked genes and skewed X-inactivation is known to be indicative of genomic alterations and less consistently genetic mutations. Indeed, it is quite a common feature in X-linked disorders [7,20,69] although some X-linked genes like *IQSEC2* are known to escape this mechanism. Skewed X-inactivation has been proposed as a protective mechanism against the mutant X chromosome and the varying degrees of X-inactivation have been attributed to milder manifestations of the disease in females and so it has been shown that there is a female phenotype in most of the XLID conditions, although X-inactivation does not always correlate with the female phenotype [70].

In spite of the absence of more candidate variants for the remaining families, XLID cannot be ruled out since other genes that have not been included in this gene panel might be the cause of XLID. Indeed, since this gene panel was designed, more genes have been reported to be associated with non-syndromic XLID [10]. This is one of the pitfalls of targeted sequencing that could be overcome by actively modifying gene panels in the basis of the current knowledge. Actually, gene panels should be dynamic and therefore should be evaluated every now and then to add new genes or remove others.

On the other hand, despite having indications such as X-linked family history that suggests XLID, ID might be of a different origin as it has been demonstrated by Sanchis-Juan et al. (2019) [17]. The family of our patient ID1208 reported that two maternal uncles of the mother had ID besides his half-brother. Nevertheless, as the grandmother is not the carrier of the *IQSEC2* missense variant, the ID of these relatives should be of different origin. Due to the ascertainment method applied, the collected clinical data are scarce and might not be as accurate. As it was shown in Table 1, most of the patients were referred before 10 years of age and had no IQ evaluation or was unknown. This could mean that they presented with global developmental delay but have not necessarily develop ID later. In line with this, not many details on comorbidities that could help in variant assessment were reported. Family history of ID was mostly based on what the parents or legal representatives of the patients reported, and no assessment was performed to determine if there were any similarities on the phenotypes they present. Being aware of this, re-contacting has been necessary to evaluate the pathogenicity of the candidate variants found and it has been possible in nearly half of them (7/16).

Segregation analysis together with RNA analysis and biochemical tests has helped in establishing the pathogenicity of the *SLC6A8* in-frame deletion. Segregation analysis itself has also been useful in excluding variants as causative-like the missense variant in *MAOA-* or identifying a plausible cause of XLID due to genotype–phenotype correlation as it has been the case of *IQSEC2* missense variant. Nevertheless, segregation analysis and genotype phenotype correlations have not been enough in determining the pathogenicity of missense variants in *SLC9A6*, *HUWE1*, *SYN1*, and *MED12*, although variants in *SLC9A6* and *MED12* seem likely pathogenic due to their co-segregation in the families. Moreover, the phenotype in ID and, consequently, XLID is highly variable and makes diagnosis difficult. Indeed, despite the nonsense variant identified in *PHF8* being pathogenic, the proband does not resemble any phenotypical features of Siderius syndrome, which makes us suspect that the phenotypical spectrum of *PHF8* variants must be expanded. To our knowledge, this case would be the first described without Siderius syndrome.

With reference to the families that could not be re-contacted, it is interesting to discuss family ID1204 because the splicing variant found (c.371-1G>C) has been classified as pathogenic (Table 2). In fact, samples of the proband and his brother got to the lab years ago from an institution for disabled people to exclude FXS. The collected clinical data indicated that they were 30 and 32 years old and had non-specific mild ID without autism. Furthermore, both had dolichocephaly and abnormal dental implantation. *UPF3B* is a gene previously implicated in XLID that encodes a protein involved in nonsense-mediated mRNA decay. The UPF3B protein is an important component of the nonsense-mediated mRNA decay surveillance machinery and it has been proposed that it may have a potential function in the regulation of the expression and degradation of various mRNAs present at the synapse [71]. A recent article [72] has added new insights into the wide variability between and within families that had been previously described. Therefore, this new family adds even more to the growing evidence of the clinical and genetic variability in neurodevelopmental disorders. As for the remaining missense candidate variants that could not further analyzed (Table 2), four of the missense variants identified in ID0919, ID1010, ID1125, and ID1304 are present in hemizygous males in population databases like GnomAD, although at very low frequency, and this could indicate that these are rare benign variants. Nevertheless, having such a low frequency, they cannot be excluded as candidate variants and were indeed prioritized using our variant filtering criteria. As for the missense variant identified in ID1011 in the *NHS* gene, despite that the variant has not been reported in population databases or the literature and is predicted to be possibly damaging by in silico predictors, mainly truncating mutations have been reported in HGMD. Mutation in *NHS* lead to either Nance Hooran Syndrome (NHS: OMIM#302350) or congenital cataracts (CTRCT40: OMIM#302200). NHS syndrome is characterized by dental anomalies, dysmorphic features, and in some cases, intellectual disability besides congenital cataracts. Our patient has severe ID, autism, epilepsy, and obesity, but cataracts have not been reported, which is typical in patients with *NHS* mutations. Similarly, a missense variant was identified in *PRPS1* gene in ID1307. This variant falls in a highly conserved residue in the ribose-phosphate diphosphokinase domain of the protein. To date, 34 missense variants have been reported in HGMD leading to four distinct syndromes: PRPS1 superactivity (OMIM#300661), X-linked Charcot-Marie-Tooth disease-5 (CMTX5: OMIM#311070), Arts syndrome (OMIM#301835), and isolated X-linked sensorineural deafness (DFNX1: OMIM#304500). All of these phenotypes described include sensoneural deafness, which was not reported in our proband that was only reported to have ID and X-linked family history. On the other hand, missense variants in *HUWE1* and *CASK* could possibly be pathogenic as the molecular evidence suggests. The variant in *HUWE1* gene is in a highly conserved residue and is predicted to be deleterious by in silico predictors. Moreover, it is located to the HECT domain where most of the mutations are clustered, which suggests that it is a likely pathogenic variant. The proband ID1206 was referred at 30 years of age with mild ID and behavioral disturbances and has a similar affected brother. Moreover, his mother has an extreme skewing X-inactivation. The missense variant in *CASK* is also in a highly conserved residue and predicted to be deleterious by in silico predictors. Furthermore, it is located on the protein kinase domain, which suggests that it is a likely pathogenic variant. In this case, the proband was referred at 27 years of age with non-syndromic mild ID and has a similar affected brother.

## 5. Conclusions

Targeted next-generation sequencing of 82 XLID genes on 61 male patients with suggestive non-syndromic XLID has demonstrated to be useful in elucidating the genetic basis of ID in some of the cases, especially in retrospective cases in which exome sequencing cannot be properly evaluated. It is important to highlight that patient and family re-contacting together with variant segregation, a more accurate description of the phenotype, and the additional tests performed have helped in reclassifying the identified variants. Indeed, *SLC6A8* in-frame variant has been reclassified as pathogenic, *IQSEC2* missense variant as likely pathogenic, and *MAOA* missense variant as likely benign. Despite variants in *MED12* and *SLC9A6* remain classified as VUS according to the ACMG criteria applied, we believe that these variants are likely pathogenic because the segregation analysis and phenotype suggest so.

With the advent of next-generation sequencing, more and more missense VUS are identified in patients with ID and XLID and therefore there is an urge to assess the pathogenicity of these variants. Phenotype correlation is not straightforward since it is highly variable in patients with ID. Therefore, functional assays are needed to assess if these variants have any impact in the protein function and consequently on the patient’s phenotype.

## Figures and Tables

**Table 1:** Description of the selected cohort of 61 patients with suggestive X-linked intellectual disability. The cohort has been divided into two groups: Index males that have suggestive X-linked inheritance (X-linked) and index males with affected brothers (Siblings).

	X Linked		Siblings	
**Total**	*n* = 47		*n* = 14	
**Age range**	2–63		2–24	
**0–10**	26	55.32%	9	64.29%
**10–20**	10	21.28%	4	28.57%
**>20**	11	23.40%	1	7.14%
**Intellectual disability**				
**Mild/Borderline**	16	34.04%	5	35.71%
**Moderate**	6	12.77%	1	7.14%
**Severe**	3	6.38%	1	7.14%
**Profound**	2	4.26%	0	
**Unknown**	20	42.55%	7	50%
**Comorbidity**				
**Macrocephaly**	1	2.13%	0	
**Microcephaly**	1	2.13%	1	7.14%
**Autism Spectrum Disorder**	14	29.79%	4	28.57%
**Hypotonia**	1	2.13%	0	
**Epilepsy**	7	14.89%	0	
**Behavioural disturbances**	5	10.64%	2	14.29%
**Previous studies**				
**Karyotype**	47	100%	14	100%
**Fragile-X**	47	100%	14	100%
**MLPA-X**	42	89.36%	14	100%
**array CGH**	15	31.91%	14	100%

**Table 2:** Variants identified in our 61-patient cohort. Variants were initially classified using InterVar software and this classification was manually adjusted after segregation analysis based on the results obtained.

Patient ID	Gene	Variant	Inheritance ^1^	Family History	GnomAD Allele Freq males	dbSNP	ClinVar	CADD ^2^	InterVar ^3^	InterVar (Manually Adjusted)
Pathogenic/likely pathogenic variants										
**ID0707**	*PHF8*	NM_001184896.1: c.252C>A; p.Tyr84*	Maternal (97.04%)	Sib-pair				36	Pathogenic (PVS1, PM2, PP3)	Pathogenic (PVS1, PM2, PP3)
**ID1204**	*UPF3B*	NM_080632.2: c.371-1G>C	Maternal (80.49%)	Sib-pair					Pathogenic (PVS1, PM2, PP3)	
**ID1122**	*SLC6A8*	NM_005629.3: c.1390_1392del GAT; p.Asp464del	Maternal (79.37%)	X-linked					Likely Pathogenic (PVS1, PM2)	Pathogenic (PS3, PM2, PM4, PP1, PP3)
**ID1208**	*IQSEC2*	NM_001111125.2: c.128G>C; p.Arg43Pro	Maternal (53.7%)	X-linked				26.2	VUS (PM2)	Likely Pathogenic (PM2, PP1, PP2, PP3, PP4)
Variants of unknown significance										
**ID0216**	*SLC9A6*	NM_001042537.1: c.316A>G; p.Met106Val	Maternal (57.09%)	X-linked				23.1	VUS (PM1, PM2, BP1)	VUS (PM1, PM2, PP1, BP1)
**ID0919**	*UPF3B*	NM_080632.2: c.1118G>A; p.Arg373His	Maternal (uninformative)	X-linked	2/66856	rs146785878	VUS	26.7	VUS (PM2, PP3)	
**ID1010**	*DLG3*	NM_021120.3: c.1424C>T; p.Ser475Leu	Maternal (91.97%)	Sib-pair	1/75937	rs953325312		31	VUS (PM1, PM2, BP1)	
**ID1011**	*NHS*	NM_198270.3: c.1270A>G; p.Arg424Gly	Maternal (uninformative)	X-linked				23.7	VUS (PM2, BP1)	
**ID1125**	*FMR1*	NM_002024.5: c.1816C>T; p.Arg606Cys	Maternal (83.18%)	X-linked	1/67871	rs782778170		34	VUS (PM1, PM2)	
**ID1128**	*HUWE1*	NM_031407.6: c.1125G>T; p.Met375Ile	Maternal (86.55%)	Sib-pair	0/41548	rs1043071474		22.6	VUS (PM1, PM2)	VUS (PM1, PM2)
**ID1205**	*CASK*	NM_003688.3: c.490G>A; p.Gly164Arg	Maternal (74.26%)	X-linked				32	VUS (PM1, PM2, PP3)	
**ID1206**	*HUWE1*	NM_031407.6: c.12209C>G; p.Ser4070Cys	Maternal (97.31%)	Sib-pair				24	VUS (PM1, PM2)	
**ID1304**	*CCDC22*	NM_014008.4: c.1388C>G; p.Ala463Gly	Maternal (54.33%)	X-linked	1/73246	rs782691732		26.9	VUS (PM2)	
**ID1307**	*PRPS1*	NM_002764.3: c.611G>A; p.Arg204His	Maternal (uninformative)	X-linked		rs1169615098		24	VUS (PM1, PM2, PP2)	
**ID1402**	*SYN1*	NM_006950.3: c.796G>A; p.Val266Met	Maternal (69.45%)	X-linked		rs1327735600		26.4	VUS (PM1, PM2)	VUS (PM1, PM2, PP3)
**ID1405**	*MED12*	NM_005120.2: c.5009C>T; p.Ser1670Phe	Maternal (95.36%)	X-linked				33	VUS (PM2)	VUS (PM2, PP1, PP3)
Benign/likely benign variants										
**ID1208**	*MAOA*	NM_000240.3: c.617G>A; p.Arg206Gln	Maternal (53.7%)	X-linked		rs1218703391		31	VUS (PM1, PM2, PP3)	Likely benign (PM1, PM2, PP3, BS2, BP5)

^1^ Parenthesis indicates mothers’ X-inactivation; ^2^ CADD scores are Phred-scaled and range from 1 to 99. Higher values are more likely to indicate deleterious effects; ^3^ Parenthesis indicates ACMG criteria applied for molecular variant classification using InterVar software; VUS: Variant of uncertain significance; PVS: Very strong criteria for pathogenic/likely pathogenic; PS: Strong criteria for pathogenic/likely pathogenic; PM: Moderate criteria for pathogenic/likely pathogenic; PP: Supporting criteria for pathogenic/likely pathogenic; BP: Supporting criteria for benign/likely benign.

## Supplementary Materials

The following are available online at https://www.mdpi.com/2073-4425/11/1/51/s1. Table S1. List of the 82 genes included in our XLID gene panel. Table S2. Technical sequencing data.
