# Supplementary material for: Targeted Next-Generation Sequencing in Patients with Suggestive X-Linked Intellectual Disability
Source: Genes (Basel). 2020 Jan 2;11(1):51. doi: 10.3390/genes11010051 (PMC7017351; doi:10.3390/genes11010051)
Supplement: Supplementary file 1 [file genes-11-00051-s001.zip › Genes-672756-Ibarluzea-Table_S1.pdf]

Table S1. List of the 82 genes included in our XLID gene panel. All the phenotypes that have been associated with each gene are shown. These phenotypes might include ID or not. Besides, references for which these genes were included in our gene panel are also listed.

Gene symbol	OMIM	Associated phenotype	References
**ACSL4**	300157	Mental retardation, X-linked 63 (OMIM#300387)	[1,2]
**AFF2**	300806	Mental retardation, X-linked, FRAXE type (OMIM#309548)	[3,4]
**AGTR2**	300034	Severe mental retardation, pervasive developmental disorder, and epilepsy	[5]
**ALG13**	300776	Congenital disorder of glycosylation, type Is; Epileptic encephalopathy, early infantile, 36 (OMIM#300884)	[6]
**AP1S2**	300629	Mental retardation, X-linked syndromic 5 (OMIM#304340)	[7]
**ARHGEF9**	300429	Epileptic encephalopathy, early infantile, 8 (OMIM#300607)	[8,9]
**ARX**	300382	Epileptic encephalopathy, early infantile, 1 (OMIM#308350)	[10]
		Hydranencephaly with abnormal genitalia Lissencephaly, X-linked 2 (OMIM#300215)	
		Mental retardation, X-linked 29 and others (OMIM#300419)	
		Partington syndrome (OMIM#309510)	
		Proud syndrome (OMIM#300004)	
**ATP6AP2**	300556	Parkinsonism with spasticity, X-linked (OMIM#300911)	[11]
		Mental retardation, X-linked, syndromic, Hedera type (OMIM#300423)	
**ATP7A**	300011	Menkes disease (OMIM#309400)	[12,13]
		Occipital horn syndrome (OMIM#304150)	
		Spinal muscular atrophy, distal, X-linked 3 (OMIM#300489)	
**ATRX**	300032	Alpha-thalassemia myelodysplasia syndrome, somatic (OMIM#300448)	[14,15]
		Alpha-thalassemia/mental retardation syndrome (OMIM#301040)	
		Mental retardation-hypotonic facies syndrome, X-linked (OMIM#309580)	
**BRWD3**	300553	Mental retardation, X-linked 93 (OMIM#300659)	[16]
**CASK**	300172	Mental retardation, with or without nystagmus; FG syndrome 4 (OMIM#300422)	[17]
		Mental retardation and microcephaly with pontine and cerebellar hypoplasia (OMIM#300749)	
**CCDC22**	300859	Ritscher-Schinzel syndrome 2 (OMIM#300963)	[18]
**CLIC2**	300138	Mental retardation, X-linked, syndromic 32 (OMIM#300886)	[19,20]
**CUL4B**	300304	Mental retardation, X-linked, syndromic 15 (Cabezas type) (OMIM#300354)	[21]

**DCX**	300121	Lissencephaly, X-linked; Subcortical laminal heterotopia, X-linked (OMIM#300067)	[22]
**DLG3**	300189	Mental retardation, X-linked 90 (OMIM#300850)	[23,24]
**DMD**	300377	Becker muscular dystrophy (OMIM#300376)	[25,26]
		Cardiomyopathy, dilated, 3B (OMIM#302045)	
		Duchenne muscular dystrophy (OMIM#310200)	
**FAM120C**	300741	Autism spectrum disorder	[27]
**FLNA**	300017	FG syndrome 2 (OMIM#300321)	[28]
		Cardiac valvular dysplasia, X-linked (OMIM#314400)	
		Congenital short bowel syndrome (OMIM#300048)	
		Frontometaphyseal dysplasia 1 (OMIM# 305620)	
		Heterotopia, periventricular, 1 (OMIM#300049)	
		Intestinal pseudoobstruction, neuronal (OMIM#300048)	
		Melnick-Needles syndrome (OMIM#309350)	
		Otopalatodigital syndrome, type I (OMIM#311300)	
		Otopalatodigital syndrome, type II (OMIM#304120)	
		Terminal osseous dysplasia (OMIM#300244)	
**FMR1**	309550	Fragile X syndrome (OMIM#300624)	[29–31]
		Fragile X tremor/ataxia syndrome (OMIM#300623)	
		Premature ovarian failure 1 (OMIM#311360)	
**FTSJ1**	300499	Mental retardation, X-linked 9/44 (OMIM#309549)	[32]
**GDI1**	300104	Mental retardation, X-linked 41 (OMIM#300849)	[33]
**GPC3**	300037	Simpson-Golabi-Behmel syndrome, type 1 (OMIM#312870)	[34,35]
		Wilms tumor, somatic (OMIM#194070)	
**GPC4**	300168	Simpson-Golabi-Behmel syndrome type 1 (OMIM#312870)	[35,36]
**GRIA3**	305915	Mental retardation, X-linked 94 (OMIM#300699)	[37,38]
**HCFC1**	300019	Mental retardation, X-linked 3 (methylmalonic acidemia and homocysteinemia, cblX type) (OMIM#309541)	[39]
**HDAC8**	300269	Cornelia de Lange syndrome 5 (OMIM#300882)	[40]

**HSD17B10**	300256	HSD10 mitochondrial disease (OMIM#300438)	[41,42]
**HUWE1**	300697	Mental retardation, X-linked syndromic, Turner type (OMIM#309590)	[42]
**IGBP1**	300139	Corpus callosum, agenesis of, with mental retardation, ocular coloboma and micrognathia (OMIM#300472)	[43]
**IL1RAPL1**	300206	Mental retardation, X-linked 21/34 (OMIM#300143)	[44]
**IQSEC2**	300522	Mental retardation, X-linked 1/78 (OMIM#309530)	[45,46]
**KAL1**	300836	Mild intelectual disability, hyperosmia, ectrodactyly	[47]
**KDM5C**	314690	Mental retardation, X-linked, syndromic, Claes-Jensen type (OMIM#300534)	[48–50]
**KDM6A**	300128	Kabuki syndrome 2 (OMIM#300867)	[51,52]
**KIAA2022**	300524	Mental retardation, X-linked 98 (OMIM#300912)	[53,54]
**L1CAM**	308840	Corpus callosum, partial agenesis of (OMIM#304100)	[55,56]
		CRASH syndrome; MASA syndrome (OMIM#303350)	
		Hydrocephalus due to aqueductal stenosis; Hydrocephalus with congenital idiopathic intestinal pseudoobstruction; Hydrocephalus with Hirschsprung disease (OMIM#307000)	
**MAOA**	309850	Brunner syndrome; Antisocial behavior (OMIM#300615)	[57,58]
**MECP2**	300005	Encephalopathy, neonatal severe (OMIM#300673)	[59,60]
		Mental retardation, X-linked syndromic, Lubs type (OMIM#300260)	
		Mental retardation, X-linked, syndromic 13 (OMIM#300055)	
		Rett syndrome; Rett syndrome, atypical; Rett syndrome, preserved speech variant (OMIM#312750)	
		Autism susceptibility, X-linked 3 (OMIM#300496)	
**MED12**	300188	Lujan-Fryns syndrome (OMIM# 309520)	[61]
		Ohdo syndrome, X-linked (OMIM#300895)	
		Opitz-Kaveggia syndrome (OMIM#305450)	
**MID1**	300552	Opitz GBBB syndrome, type I (OMIM#300000)	[62]
**NAA10**	300013	Microphthalmia, syndromic 1 (OMIM#309800)	[63,64]
		Ogden syndrome (OMIM#300855)	
**NDP**	300658	Exudative vitreoretinopathy 2, X-linked (OMIM#305390)	[65]
		Norrie disease (OMIM#310600)	

**NHS**	300457	Cataract 40, X-linked (OMIM#302200)	[66]
		Nance-Horan syndrome (OMIM#302350)	
**NLGN3**	300336	Asperger syndrome susceptibility, X-linked 1 (OMIM#300494)	[67]
		Autism susceptibility, X-linked 1 (OMIM#300425)	
**NLGN4X**	300427	Mental retardation, X-linked; Autism susceptibility, X-linked 2 (OMIM#300495)	[67]
		Asperger syndrome susceptibility, X-linked 2 (OMIM#300497)	
**NSDHL**	300275	CHILD syndrome (OMIM#308050)	[68,69]
		CK syndrome (OMIM#300831)	
**OFD1**	300170	Retinitis pigmentosa 23 (OMIM#300424)	[70–72]
		Joubert syndrome 10 (OMIM#300804)	
		Orofaciodigital syndrome I (OMIM#311200)	
		Simpson-Golabi-Behmel syndrome, type 2 (OMIM#300209)	
**OPHN1**	300127	Mental retardation, X-linked, with cerebellar hypoplasia and distinctive facial appearance (OMIM#300486)	[73]
**PAK3**	300142	Mental retardation, X-linked 30/47 (OMIM#300558)	[74]
**PHF6**	300414	Borjeson-Forssman-Lehmann syndrome (OMIM#301900)	[75]
**PHF8**	300560	Mental retardation syndrome, X-linked, Siderius type (OMIM#300263)	[76]
**PLP1**	300401	Pelizaeus-Merzbacher disease (OMIM#312080)	[77]
		Spastic paraplegia 2, X-linked (OMIM#312920)	
**PQBP1**	300463	Renpenning syndrome (OMIM#309500)	[78,79]
**PRPS1**	311850	Arts syndrome (OMIM#301835)	[80]
		Charcot-Marie-Tooth disease, X-linked recessive, 5 (OMIM#311070)	
		Deafness, X-linked 1 (OMIM#304500)	
		Gout, PRPS-related; Phosphoribosylpyrophosphate synthetase superactivity (OMIM#300661)	
**PTCHD1**	300828	Autism, susceptibility to, X-linked 4 (OMIM#300830)	[81,82]
**RAB39B**	300774	Mental retardation, X-linked 72 (OMIM#300271)	[83]
		Waisman syndrome (OMIM#311510)	
**RBM10**	300080	TARP syndrome (OMIM#311900)	[84,85]

**RBMX**	300199	Mental retardation, X-linked, syndromic 11, Shashi type (OMIM#300238)	[86]
**RNF113A**	300951	Trichothiodystrophy 5, nonphotosensitive (OMIM#300953)	[87]
**RPL10**	312173	Mental retardation, X-linked, syndromic, 35 (OMIM#300998)	[88,89]
		Autism, susceptibility to, X-linked 5 (OMIM#300847)	
**RPS6KA3**	300075	Coffin-Lowry syndrome (OMIM#303600)	[90]
		Mental retardation, X-linked 19 (OMIM#300844)	
**SLC16A2**	300095	Allan-Herndon-Dudley syndrome (OMIM#300523)	[91,92]
**SLC6A8**	300036	Cerebral creatine deficiency syndrome 1 (OMIM#300352)	[93]
**SLC9A6**	300231	Mental retardation, X-linked syndromic, Christianson type (OMIM#300243)	[94]
**SMARCA1**	300012	Intellectual disability, microcephaly and spasticity (Coffin-Siris like phenotype)	[95]
**SMC1A**	300040	Cornelia de Lange syndrome 2 (OMIM#300590)	[96]
**SMS**	300105	Mental retardation, X-linked, Snyder-Robinson type (OMIM#309583)	[97]
**SRPX2**	300642	Rolandic epilepsy, mental retardation, and speech dyspraxia (OMIM#300643)	[98]
**SYN1**	313440	Epilepsy, X-linked, with variable learning disabilities and behavior disorders (OMIM#300491)	[99]
**SYP**	313475	Mental retardation, X-linked 96 (OMIM#300802)	[100]
**TAF1**	313650	Dystonia-Parkinsonism, X-linked (OMIM#314250)	[101]
		Mental retardation, X-linked, syndromic 33 (OMIM#300966)	
**TSPAN7**	300096	Mental retardation, X-linked 58 (OMIM#300210)	[102]
**UBE2A**	312180	Mental retardation, X-linked syndromic, Nascimento-type (OMIM#300860)	[103]
**UPF3B**	300298	Mental retardation, X-linked, syndromic 14 (OMIM#300676)	[104,105]
**USP9X**	300072	Mental retardation, X-linked 99 (OMIM#300919)	[106]
		Mental retardation, X-linked 99, syndromic, female-restricted (OMIM#300968)	
**WDR45**	300526	Neurodegeneration with brain iron accumulation 5 (OMIM#300894)	[107]
**ZC4H2**	300897	Wieacker-Wolff syndrome (OMIM#314580)	[108]
**ZDHHC15**	300576	Severe non-syndromic intellectual disability (female)	[109,110]
**ZDHHC9**	300646	Mental retardation, X-linked syndromic, Raymond type (OMIM#300799)	[111]
**ZNF711**	314990	Mental retardation, X-linked 97 (OMIM#300803)	[100]

References

1. Meloni, I.; Muscettola, M.; Raynaud, M.; Longo, I.; Bruttini, M.; Moizard, M.P.; Gomot, M.; Chelly, J.; Des Portes, V.; Fryns, J.P.; et al. FACL4, encoding fatty acid-CoA ligase 4, is mutated in nonspecific X-linked mental retardation. *Nat. Genet.* **2002**, *30*, 436–440.

2. Longo, I.; Schwartz, C.; Renieri, A.; Froyen, G.; Frints, S.G.M.; Fryns, J.P.; Meloni, I.; Pescucci, C.; Ariani, F.; Borghgraef, M.; et al. A third MRX family (MRX68) is the result of mutation in the long chain fatty acid-CoA ligase 4 (FACL4) gene: Proposal of a rapid enzymatic assay for screening mentally retarded patients. *J. Med. Genet.* **2003**, *40*, 11–17.

3. Stettner, G.M.; Shoukier, M.; Höger, C.; Brockmann, K.; Auber, B. Familial intellectual disability and autistic behavior caused by a small FMR2 gene deletion. *Am. J. Med. Genet. Part A* **2011**, *155*, 2003–2007.

4. Mondal, K.; Ramachandran, D.; Patel, V.C.; Hagen, K.R.; Bose, P.; Cutler, D.J.; Zwick, M.E. Excess variants in AFF2 detected by massively parallel sequencing of males with autism spectrum disorder. *Hum. Mol. Genet.* **2012**, *21*, 4356–4364.

5. Takeshita, E.; Nakagawa, E.; Nakatani, K.; Sasaki, M.; Goto, Y.I. Novel AGTR2 missense mutation in a Japanese boy with severe mental retardation, pervasive developmental disorder, and epilepsy. *Brain Dev.* **2012**, *34*, 776–779.

6. Bissar-Tadmouri, N.; Donahue, W.L.; Al-Gazali, L.; Nelson, S.F.; Bayrak-Toydemir, P.; Kantarci, S. X chromosome exome sequencing reveals a novel ALG13 mutation in a nonsyndromic intellectual disability family with multiple affected male siblings. *Am. J. Med. Genet. Part A* **2014**, *164*, 164–169.

7. Tarpey, P.S.; Stevens, C.; Teague, J.; Edkins, S.; O'Meara, S.; Avis, T.; Barthorpe, S.; Buck, G.; Butler, A.; Cole, J.; et al. Mutations in the gene encoding the sigma 2 subunit of the adaptor protein 1 complex, AP1S2, cause X-linked mental retardation. *Am. J. Hum. Genet.* **2006**, *79*, 1119–1124.

8. Harvey, K.; Duguid, I.C.; Alldred, M.J.; Beatty, S.E.; Ward, H.; Keep, N.H.; Lingenfelter, S.E.; Pearce, B.R.; Lundgren, J.; Owen, M.J.; et al. The GDP-GTP exchange factor collybistin: An essential determinant of neuronal gephyrin clustering. *J. Neurosci.* **2004**, *24*, 5816–5826.

9. Shimojima, K.; Sugawara, M.; Shichiji, M.; Mukaida, S.; Takayama, R.; Imai, K.; Yamamoto, T. Loss-of-function mutation of collybistin is responsible for X-linked mental retardation associated with epilepsy. *J. Hum. Genet.* **2011**, *56*, 561–565.

10. Shoubridge, C.; Fullston, T.; Gécz, J. ARX spectrum disorders: Making inroads into the molecular pathology. *Hum. Mutat.* **2010**, *31*, 889–900.

11. Gupta, H. V.; Vengoechea, J.; Sahaya, K.; Virmani, T. A splice site mutation in ATP6AP2 causes X-linked intellectual disability, epilepsy, and parkinsonism. *Park. Relat. Disord.* **2015**, *21*, 1473–1475.

12. Kaler, S.G.; Gallo, L.K.; Proud, V.K.; Percy, A.K.; Mark, Y.; Segal, N.A.; Goldstein, D.S.; Holmes, C.S.; Gahl, W.A. Occipital horn syndrome and a mild Menkes phenotype associated with splice site mutations at the MNK locus. *Nat. Genet.* **1994**, *8*, 195–202.

13. Tümer, Z. An Overview and Update of ATP7A Mutations Leading to Menkes Disease and Occipital Horn Syndrome. *Hum. Mutat.* **2013**, *34*, 417–429.

14. Yntema, H.G.; Poppelaars, F.A.; Derksen, E.; Oudakker, A.R.; Van Roosmalen, T.; Jacobs, A.; Obbema, H.; Brunner, H.G.; Hamel, B.C.J.; Van Bokhoven, H. Expanding phenotype of XNP mutations: Mild to moderate mental retardation. *Am. J. Med. Genet.* **2002**, *110*, 243–247.

15. Moncini, S.; Bedeschi, M.F.; Castronovo, P.; Crippa, M.; Calvello, M.; Garghentino, R.R.; Scuvera, G.; Finelli, P.; Venturin, M. ATRX mutation in two adult brothers with non-specific moderate intellectual disability identified by exome sequencing. *Meta Gene* **2013**, *1*, 102–108.

16. Field, M.; Tarpey, P.S.; Smith, R.; Edkins, S.; O'Meara, S.; Stevens, C.; Tofts, C.; Teague, J.; Butler, A.; Dicks, E.; et al. Mutations in the BRWD3 gene cause X-linked mental retardation associated with macrocephaly. *Am. J. Hum. Genet.* **2007**, *81*, 367–374.

17. Hackett, A.; Tarpey, P.S.; Licata, A.; Cox, J.; Whibley, A.; Boyle, J.; Rogers, C.; Grigg, J.; Partington, M.; Stevenson, R.E.; et al. CASK mutations are frequent in males and cause X-linked nystagmus and variable XLMR phenotypes. *Eur. J. Hum. Genet.* **2010**, *18*, 544–552.

18. Voineagu, I.; Huang, L.; Winden, K.; Lazaro, M.; Haan, E.; Nelson, J.; McGaughran, J.; Nguyen, L.; Friend, K.; Hackett, A.; et al. CCDC22: a novel candidate gene for syndromic X-linked intellectual disability. *Mol Psychiatry* **2012**, *17*, 4–7.

19. Witham, S.; Takano, K.; Schwartz, C.; Alexov, E. A missense mutation in CLIC2 associated with intellectual disability is predicted by in silico modeling to affect protein stability and dynamics. *Proteins Struct. Funct. Bioinforma.* **2011**, *79*, 2444–2454.

20. Takano, K.; Liu, D.; Tarpey, P.; Gallant, E.; Lam, A.; Witham, S.; Alexov, E.; Chaubey, A.; Stevenson, R.E.; Schwartz, C.E.; et al. An x-linked channelopathy with cardiomegaly due to a CLIC2 mutation enhancing ryanodine receptor channel activity. *Hum. Mol. Genet.* **2012**, *21*, 4497–4507.

21. Tarpey, P.S.; Raymond, F.L.; O'Meara, S.; Edkins, S.; Teague, J.; Butler, A.; Dicks, E.; Stevens, C.; Tofts, C.; Avis, T.; et al. Mutations in CUL4B, which encodes a ubiquitin E3 ligase subunit, cause an X-linked mental retardation syndrome associated with aggressive outbursts, seizures, relative macrocephaly, central obesity, hypogonadism, pes cavus, and tremor. *Am. J. Hum. Genet.* **2007**, *80*, 345–352.

22. Guerrini, R.; Moro, F.; Andermann, E.; Hughes, E.; D'Agostino, D.; Carrozzo, R.; Bernasconi, A.; Flinter, F.; Parmeggiani, L.; Volzone, A.; et al. Nonsyndromic mental retardation and cryptogenic epilepsy in women with doublecortin gene mutations. *Ann. Neurol.* **2003**, *54*, 30–37.

23. Tarpey, P.; Parnau, J.; Blow, M.; Woffendin, H.; Bignell, G.; Cox, C.; Cox, J.; Davies, H.; Edkins, S.; Holden, S.; et al. Mutations in the DLG3 gene cause

nonsyndromic X-linked mental retardation. *Am J Hum Genet* **2004**, *75*, 318–324.

24. Zanni, G.; Van Esch, H.; Bensalem, A.; Saillour, Y.; Poirier, K.; Castelnau, L.; Ropers, H.H.; de Brouwer, A.P.M.; Laumonnier, F.; Fryns, J.P.; et al. A novel mutation in the DLG3 gene encoding the synapse-associated protein 102 (SAP102) causes non-syndromic mental retardation. *Neurogenetics* **2010**, *11*, 251–255.

25. Srour, M.; Bejjani, B.A.; Rorem, E.A.; Hall, N.; Shaffer, L.G.; Shevell, M.I. An Instructive Case of an 8-Year-Old Boy With Intellectual Disability. *Semin. Pediatr. Neurol.* **2008**, *15*, 154–155.

26. De Brouwer, A.P.; Nabuurs, S.B.; Verhaart, I.E.; Oudakker, A.R.; Hordijk, R.; Yntema, H.G.; Hordijk-Hos, J.M.; Voesenek, K.; De Vries, B.B.; Van Essen, T.; et al. A 3-base pair deletion, c.9711-9713del, in DMD results in intellectual disability without muscular dystrophy. *Eur. J. Hum. Genet.* **2014**, *22*, 480–485.

27. De Wolf, V.; Crepel, A.; Schuit, F.; van Lommel, L.; Ceulemans, B.; Steyaert, J.; Seuntjens, E.; Peeters, H.; Devriendt, K. A complex Xp11.22 deletion in a patient with syndromic autism: Exploration of FAM120C as a positional candidate gene for autism. *Am. J. Med. Genet. Part A* **2014**, *164*, 3035–3041.

28. Robertson, S.P. Filamin A: Phenotypic diversity. *Curr. Opin. Genet. Dev.* **2005**, *15*, 301–307.

29. Collins, S.C.; Bray, S.M.; Suhl, J.A.; Cutler, D.J.; Coffee, B.; Zwick, M.E.; Warren, S.T. Identification of novel FMR1 variants by massively parallel sequencing in developmentally delayed males. *Am. J. Med. Genet. Part A* **2010**, *152 A*, 2512–2520.

30. Myrick, L.K.; Deng, P.Y.; Hashimoto, H.; Oh, Y.M.; Cho, Y.; Poidevin, M.J.; Suhl, J.A.; Visootsak, J.; Cavalli, V.; Jin, P.; et al. Independent role for presynaptic FMRP revealed by an FMR1 missense mutation associated with intellectual disability and seizures. *Proc Natl Acad Sci U S A* **2015**, *112*, 949–956.

31. Myrick, L.K.; Nakamoto-Kinoshita, M.; Lindor, N.M.; Kirmani, S.; Cheng, X.; Warren, S.T. Fragile X syndrome due to a missense mutation. *Eur J Hum Genet* **2014**, *22*, 1185–1189.

32. Freude, K.; Hoffmann, K.; Jensen, L.R.; Delatycki, M.B.; Des Portes, V.; Moser, B.; Hamel, B.; Van Bokhoven, H.; Moraine, C.; Fryns, J.P.; et al. Mutations in the FTSJ1 gene coding for a novel S-adenosylmethionine-binding protein cause nonsyndromic X-linked mental retardation. *Am. J. Hum. Genet.* **2004**, *75*, 305–309.

33. D'Adamo, P.; Menegon, A.; Nigro, C. Lo; Grasso, M.; Gulisano, M.; Tamanini, F.; Bienvenu, T.; Gedeon, A.K.; Oostra, B.; Wu, S.K.; et al. Mutations in GDI1 are responsible for X-linked non-specific mental retardation. *Nat. Genet.* **1998**, *19*, 134–139.

34. Veugelers, M.; De Cat, B.; Muyldermans, S.Y.; Reekmans, G.; Delande, N.; Frints, S.; Legius, E.; Fryns, J.-P.; Schrander-Stumpel, C.; Weidle, B.; et al. Mutational analysis of the GPC3/GPC4 glypican gene cluster on Xq26 in patients with Simpson-Golabi-Behmel syndrome: identification of loss-of-function mutations in the GPC3 gene. *Hum. Mol. Genet.* **2000**, *9*, 1321–1328.

35. Cottereau, E.; Mortemousque, I.; Moizard, M.P.; Bürglen, L.; Lacombe, D.; Gilbert-Dussardier, B.; Sigaudy, S.; Boute, O.; David, A.; Faivre, L.; et al. Phenotypic spectrum of simpson-golabi-behmel syndrome in a series of 42 cases with a mutation in GPC3 and review of the literature. *Am. J. Med. Genet. Part C Semin. Med. Genet.* **2013**, *163*, 92–105.

36. Waterson, J.; Stockley, T.L.; Segal, S.; Golabi, M. Novel duplication in glypican-4 as an apparent cause of Simpson-Golabi-Behmel syndrome. *Am. J. Med. Genet. Part A* **2010**, *152 A*, 3179–3181.

37. Wu, Y.; Arai, A.C.; Rumbaugh, G.; Srivastava, A.K.; Turner, G.; Hayashi, T.; Suzuki, E.; Jiang, Y.; Zhang, L.; Rodriguez, J.; et al. Mutations in ionotropic AMPA receptor 3 alter channel properties and are associated with moderate cognitive impairment in humans. *Proc. Natl. Acad. Sci. U. S. A.* **2007**, *104*, 18163–18168.

38. Philips, A.K.; Sirén, A.; Avela, K.; Somer, M.; Peippo, M.; Ahvenainen, M.; Doagu, F.; Arvio, M.; Kääriäinen, H.; Van Esch, H.; et al. X-exome sequencing in Finnish families with Intellectual Disability - Four novel mutations and two novel syndromic phenotypes. *Orphanet J. Rare Dis.* **2014**, *9*, 1–13.

39. Huang, L.; Jolly, L.A.; Willis-Owen, S.; Gardner, A.; Kumar, R.; Douglas, E.; Shoubridge, C.; Wieczorek, D.; Tzschach, A.; Cohen, M.; et al. A noncoding, regulatory mutation implicates HCFC1 in nonsyndromic intellectual disability. *Am. J. Hum. Genet.* **2012**, *91*, 694–702.

40. Kaiser, F.J.; Ansari, M.; Braunholz, D.; Gil-Rodríguez, M.C.; Decroos, C.; Wilde, J.J.; Fincher, C.T.; Kaur, M.; Bando, M.; Amor, D.J.; et al. Loss-of-function HDAC8 mutations cause a phenotypic spectrum of Cornelia de Lange syndrome-like features, ocular hypertelorism, large fontanelle and X-linked inheritance. *Hum. Mol. Genet.* **2014**, *23*, 2888–2900.

41. Lenski, C.; Kooy, R.F.; Reyniers, E.; Loessner, D.; Wanders, R.J.A.; Winnepenninckx, B.; Hellebrand, H.; Engert, S.; Schwartz, C.E.; Meindl, A.; et al. The reduced expression of the HADH2 protein causes X-linked mental retardation, choreoathetosis, and abnormal behavior. *Am. J. Hum. Genet.* **2007**, *80*, 372–377.

42. Froyen, G.; Corbett, M.; Vandewalle, J.; Jarvela, I.; Lawrence, O.; Meldrum, C.; Bauters, M.; Govaerts, K.; Vandeleur, L.; Van Esch, H.; et al. Submicroscopic Duplications of the Hydroxysteroid Dehydrogenase HSD17B10 and the E3 Ubiquitin Ligase HUWE1 Are Associated with Mental Retardation. *Am. J. Hum. Genet.* **2008**, *82*, 432–443.

43. Graham Jr., J.M.; Wheeler, P.; Tackels-Horne, D.; Lin, A.E.; Hall, B.D.; May, M.; Short, K.M.; Schwartz, C.E.; Cox, T.C. A new X-linked syndrome with agenesis of the corpus callosum, mental retardation, coloboma, micrognathia, and a mutation in theAlpha 4 gene at Xq13. *Am. J. Med. Genet.* **2003**, *123A*, 37–44.

44. Carrié, A.; Jun, L.; Bienvenu, T.; Vinet, M.C.; McDonell, N.; Couvert, P.; Zemni, R.; Cardona, A.; Van Buggenhout, G.; Frints, S.; et al. A new member of the IL-1 receptor family highly expressed in hippocampus and involved in X-linked mental retardation. *Nat. Genet.* **1999**, *23*, 25–31.

45. Shoubridge, C.; Tarpey, P.S.; Abidi, F.; Ramsden, S.L.; Rujirabanjerd, S.; Murphy, J.A.; Boyle, J.; Shaw, M.; Gardner, A.; Proos, A.; et al. Mutations in the guanine nucleotide exchange factor gene IQSEC2 cause nonsyndromic intellectual disability. *Nat. Genet.* **2010**, *42*, 486–488.

46. Gandomi, S.K.; Farwell Gonzalez, K.D.; Parra, M.; Shahmirzadi, L.; Mancuso, J.; Pichurin, P.; Temme, R.; Dugan, S.; Zeng, W.; Tang, S. Diagnostic exome sequencing identifies two novel IQSEC2 mutations associated with X-linked intellectual disability with seizures: Implications for genetic counseling and clinical diagnosis. *J. Genet. Couns.* **2014**, *23*, 289–298.

47. Sowińska-Seidler, A.; Piwecka, M.; Olech, E.; Socha, M.; Latos-Bieleńska, A.; Jamsheer, A. Hyperosmia, ectrodactyly, mild intellectual disability, and other defects in a male patient with an X-linked partial microduplication and overexpression of the KAL1 gene. *J. Appl. Genet.* **2015**, *56*, 177–184.

48. Jensen, L.R.; Amende, M.; Gurok, U.; Moser, B.; Gimmel, V.; Tzschach, A.; Janecke, A.R.; Tariverdian, G.; Chelly, J.; Fryns, J.P.; et al. Mutations in the JARID1C gene, which is involved in transcriptional regulation and chromatin remodeling, cause X-linked mental retardation. *Am. J. Hum. Genet.* **2005**, *76*, 227–236.

49. Tzschach, A.; Lenzner, S.; Moser, B.; Reinhardt, R.; Chelly, J.; Fryns, J.P.; Kleefstra, T.; Raynaud, M.; Turner, G.; Ropers, H.H.; et al. Novel JARID1C/SMCX mutations in patients with X-linked mental retardation. *Hum. Mutat.* **2006**, *27*, 389.

50. Adegbola, A.; Gao, H.; Sommer, S.; Browning, M. A Novel Mutation in JARID1C/SMCX in a Patient With Autism Spectrum Disorder (ASD). *Am. J. Med. Genet. Part A* **2008**, *146A*, 505–511.

51. Lederer, D.; Grisart, B.; Digilio, M.C.; Benoit, V.; Crespin, M.; Ghariani, S.C.; Maystadt, I.; Dallapiccola, B.; Verellen-Dumoulin, C. Deletion of KDM6A, a histone demethylase interacting with MLL2, in three patients with kabuki syndrome. *Am. J. Hum. Genet.* **2012**, *90*, 119–124.

52. Miyake, N.; Mizuno, S.; Okamoto, N.; Ohashi, H.; Shiina, M.; Ogata, K.; Tsurusaki, Y.; Nakashima, M.; Saitsu, H.; Niikawa, N.; et al. KDM6A Point Mutations Cause Kabuki Syndrome. *Hum. Mutat.* **2013**, *34*, 108–110.

53. Van Maldergem, L.; Hou, Q.; Kalscheuer, V.M.; Rio, M.; Doco-Fenzy, M.; Medeira, A.; de Brouwer, A.P.M.; Cabrol, C.; Haas, S.A.; Cacciagli, P.; et al.

Loss of function of KIAA2022 causes mild to severe intellectual disability with an autism spectrum disorder and impairs neurite outgrowth. *Hum. Mol. Genet.* **2013**, *22*, 3306–3314.

54. Kuroda, Y.; Ohashi, I.; Naruto, T.; Ida, K.; Enomoto, Y.; Saito, T.; Nagai, J.I.; Wada, T.; Kurosawa, K. Delineation of the KIAA2022 mutation phenotype: Two patients with X-linked intellectual disability and distinctive features. *Am. J. Med. Genet. Part A* **2015**, *167*, 1349–1353.

55. Rosenthal, A.; Jouet, M.; Kenwrick, S. Aberrant splicing of neural cell adhesion molecule L1 mRNA in a family with X-linked hydrocephalus [published erratum appears in Nat Genet 1993 Mar;3(3):273]. *Nat. Genet.* **1992**, *2*, 107–112.

56. Jouet, M.; Rosenthal, A.; Armstrong, G.; Macfarlane, J.; Stevenson, R.; Paterson, J.; Metzenberg, A.; Ionasescu, V.; Temple, K.; Kenwrick, S. X–linked spastic paraplegia (SPG1), MASA syndrome and X–linked hydrocephalus result from mutations in the L1 gene. *Nat. Genet.* **1994**, *7*, 402–407.

57. Brunner, H.G.; Nelen, M.R.; van Zandvoort, P.; Abeling, N.G.G.M.; van Gennip, A.H.; Wolters, E.C.; Kuiper, M.A.; Ropers, H.H.; Van Oost, B.A. X-linked borderline mental retardation with prominent behavioral disturbance: Phenotype, genetic localization, and evidence for disturbed monoamine metabolism. *Am. J. Hum. Genet.* **1993**, *52*, 1032–1039.

58. Piton, A.; Poquet, H.; Redin, C.; Masurel, A.; Lauer, J.; Muller, J.; Thevenon, J.; Herenger, Y.; Chancenotte, S.; Bonnet, M.; et al. 20 ans après: A second mutation in MAOA identified by targeted high-throughput sequencing in a family with altered behavior and cognition. *Eur. J. Hum. Genet.* **2014**, *22*, 776–783.

59. Orrico, A.; Lam, C.W.; Galli, L.; Dotti, M.T.; Hayek, G.; Tong, S.F.; Poon, P.M.K.; Zappella, M.; Federico, A.; Sorrentino, V. MECP2 mutation in male patients with non-specific X-linked mental retardation. *FEBS Lett.* **2000**, *481*, 285–288.

60. Villard, L. MECP2 mutations in males. *J. Med. Genet.* **2007**, *44*, 417–423.

61. Lesca, G.; Moizard, M.; Bussy, G.; Boggio, D.; Hu, H.; Haas, S.A.; Ropers, H.; Kalscheuer, V.M.; Portes, V. Des; Labalme, A.; et al. Clinical and Neurocognitive Characterization of a Family With a Novel MED12 Gene Frameshift Mutation. **2013**.

62. Quaderi, N.A.; Schweiger, S.; Gaudenz, K.; Franco, B.; Rugarli, E.I.; Berger, W.; Feldman, G.J.; Volta, M.; Andolfi, G.; Gilgenkrantz, S.; et al. Opitz G/BBB syndrome, a defect of midline development, is due to mutations in a new RING finger gene on Xp22. *Nat. Genet.* **1997**, *17*, 285–291.

63. Casey, J.P.; Støve, S.I.; McGorrian, C.; Galvin, J.; Blenski, M.; Dunne, A.; Ennis, S.; Brett, F.; King, M.D.; Arnesen, T.; et al. NAA10 mutation causing a novel intellectual disability syndrome with Long QT due to N-terminal acetyltransferase impairment. *Sci. Rep.* **2015**, *5*, 1–14.

64. Popp, B.; Støve, S.I.; Endele, S.; Myklebust, L.M.; Hoyer, J.; Sticht, H.; Azzarello-Burri, S.; Rauch, A.; Arnesen, T.; Reis, A. De novo missense mutations in the NAA10 gene cause severe non-syndromic developmental delay in males and females. *Eur. J. Hum. Genet.* **2015**, *23*, 602–609.

65. Berger, W.; De Pol, D. Van; Warburg, M.; Gal, A.; Bleeker-wagemakers, L.; De Silva, H.; Meindl, A.; Meitinger, T.; Cremers, F.; Ropers, H.H. Mutations in the candidate gene for norrie disease. *Hum. Mol. Genet.* **1992**, *1*, 461–465.

66. Burdon, K.P.; McKay, J.D.; Sale, M.M.; Russell-Eggitt, I.M.; Mackey, D.A.; Wirth, M.G.; Elder, J.E.; Nicoll, A.; Clarke, M.P.; FitzGerald, L.M.; et al. Mutations in a Novel Gene, NHS, Cause the Pleiotropic Effects of Nance-Horan Syndrome, Including Severe Congenital Cataract, Dental Anomalies, and Mental Retardation. *Am. J. Hum. Genet.* **2003**, *73*, 1120–1130.

67. Jamain, S.; Quach, H.; Betancur, C.; Råstam, M.; Colineaux, C.; Gillberg, C.; Soderstrom, H.; Giros, B.; Leboyer, M.; Gillberg, C.; et al. Mutations of the X-linked genes encoding neuroligins NLGN3 and NLGN4 are associated with autism. *Nat. Genet.* **2003**.

68. König, A.; Happle, R.; Bornholdt, D.; Engel, H.; Grzeschik, K.H. Mutations in the NSDHL gene, encoding a 3β-hydroxysteroid dehydrogenase, cause CHILD syndrome. *Am. J. Med. Genet.* **2000**, *90*, 339–346.

69. McLarren, K.W.; Severson, T.M.; Du Souich, C.; Stockton, D.W.; Kratz, L.E.; Cunningham, D.; Hendson, G.; Morin, R.D.; Wu, D.; Paul, J.E.; et al. Hypomorphic temperature-sensitive alleles of NSDHL cause CK syndrome. *Am. J. Hum. Genet.* **2010**, *87*, 905–914.

70. Ferrante, M.I.; Giorgio, G.; Feather, S.A.; Bulfone, A.; Wright, V.; Ghiani, M.; Selicorni, A.; Gammaro, L.; Scolari, F.; Woolf, A.S.; et al. Identification of the gene for oral-facial-digital type 1 syndrome. *Am. J. Hum. Genet.* **2001**, *68*, 569–576.

71. Budny, B.; Chen, W.; Omran, H.; Fliegauf, M.; Tzschach, A.; Wisniewska, M.; Jensen, L.R.; Raynaud, M.; Shoichet, S.A.; Badura, M.; et al. A novel X-linked recessive mental retardation syndrome comprising macrocephaly and ciliary dysfunction is allelic to oral-facial-digital type I syndrome. *Hum. Genet.* **2006**, *120*, 171–178.

72. Coene, K.L.M.; Roepman, R.; Doherty, D.; Afroze, B.; Kroes, H.Y.; Letteboer, S.J.F.; Ngu, L.H.; Budny, B.; van Wijk, E.; Gorden, N.T.; et al. OFD1 Is Mutated in X-Linked Joubert Syndrome and Interacts with LCA5-Encoded Lebercilin. *Am. J. Hum. Genet.* **2009**, *85*, 465–481.

73. Billuart, P.; Bienvenu, T.; Roncet, N.; Des Portes, V.; Vinet, M.C.; Zemni, R.; Crollius, H.R.; Carrié, A.; Fauchereau, F.; Cherry, M.; et al. Oligophrenin-1 encodes a rhoGAP protein involved in X-linked mental retardation. *Nature* **1998**, *392*, 923–926.

74. Allen, K.M.; Gleeson, J.G.; Bagrodia, S.; Partington, M.W.; MacMillan, J.C.; Cerione, R.A.; Mulley, J.C.; Walsh, C.A. PAK3 mutation in nonsyndromic X-linked mental retardation. *Nat. Genet.* **1998**, *20*, 25–30.

75. Lower, K.M.; Turner, G.; Kerr, B.A.; Mathews, K.D.; Shaw, M.A.; Gedeon, Á.K.; Schelley, S.; Hoyme, H.E.; White, S.M.; Delatycki, M.B.; et al. Mutations in PHF6 are associated with Börjeson-Forssman-Lehmann syndrome. *Nat. Genet.* **2002**, *32*, 661–665.

76. Laumonnier, F.; Holbert, S.; Ronce, N.; Faravelli, F.; Lenzner, S.; Schwartz, C.E.; Lespinasse, J.; Van Esch, H.; Lacombe, D.; Goizet, C.; et al. Mutations in PHF8 are associated with X linked mental retardation and cleft lip/cleft palate. *J. Med. Genet.* **2005**, *42*, 780–786.

77. Saugier-Veber, P.; Munnich, A.; Bonneau, D.; Rozet, J.-M.; Le Merrer, M.; Gil, R.; Boespflug-Tanguy, O. X-linked spastic paraplegia and Pelizaeus-Merzbacher disease are allelic disorders at the proteolipid protein locus Pascale. *Nat. Gene* **1994**, *6*, 257–262.

78. Kalscheuer, V.M.; Freude, K.; Musante, L.; Jensen, L.R.; Yntema, H.G.; Gécz, J.; Sefiani, A.; Hoffmann, K.; Moser, B.; Haas, S.; et al. Mutations in the polyglutamine binding protein 1 gene cause X-linked mental retardation. *Nat. Genet.* **2003**, *35*, 313–315.

79. Lenski, C.; Abidi, F.; Meindl, A.; Gibson, A.; Platzer, M.; Kooy, R.F.; Lubs, H.A.; Stevenson, R.E.; Ramser, J.; Schwartz, C.E. Novel Truncating Mutations in the Polyglutamine Tract Binding Protein 1 Gene (PQBP1) Cause Renpenning Syndrome and X-Linked Mental Retardation in Another Family with Microcephaly. *Am. J. Hum. Genet.* **2004**, *74*, 777–780.

80. Mittal, R.; Patel, K.; Mittal, J.; Chan, B.; Yan, D.; Grati, M.; Liu, X.Z. Association of PRPS1 Mutations with Disease Phenotypes. *Dis Markers* **2015**, *2015*, 127013.

81. Noor, A.; Whibley, A.; Marshall, C.R.; Gianakopoulos, P.J.; Piton, A.; Carson, A.R.; Orlic-Milacic, M.; Lionel, A.C.; Sato, D.; Pinto, D.; et al. Disruption at the PTCHD1 locus on Xp22.11 in autism spectrum disorder and intellectual disability. *Sci. Transl. Med.* **2010**, *2*.

82. Chaudhry, A.; Noor, A.; Degagne, B.; Baker, K.; Bok, L.A.; Brady, A.F.; Chitayat, D.; Chung, B.H.; Cytrynbaum, C.; Dyment, D.; et al. Phenotypic spectrum associated with PTCHD1 deletions and truncating mutations includes intellectual disability and autism spectrum disorder. *Clin. Genet.* **2015**, *88*, 224–233.

83. Giannandrea, M.; Bianchi, V.; Mignogna, M.L.; Sirri, A.; Carrabino, S.; D'Elia, E.; Vecellio, M.; Russo, S.; Cogliati, F.; Larizza, L.; et al. Mutations in the Small GTPase Gene RAB39B Are Responsible for X-linked Mental Retardation Associated with Autism, Epilepsy, and Macrocephaly. *Am. J. Hum. Genet.* **2010**, *86*, 185–195.

84. Johnston, J.J.; Teer, J.K.; Cherukuri, P.F.; Hansen, N.F.; Loftus, S.K.; Chong, K.; Mullikin, J.C.; Biesecker, L.G. Massively Parallel Sequencing of Exons on the X Chromosome Identifies RBM10 as the Gene that Causes a Syndromic Form of Cleft Palate. *Am. J. Hum. Genet.* **2010**, *86*, 743–748.

85. Johnston, J.J.; Sapp, J.C.; Curry, C.; Horton, M.; Leon, E.; Cusmano-Ozog, K.; Dobyns, W.B.; Hudgins, L.; Zackai, E.; Biesecker, L.G. Expansion of the

TARP syndrome phenotype associated with de novo mutations and mosaicism. *Am. J. Med. Genet. Part A* **2014**, *164*, 120–128.

86. Shashi, V.; Xie, P.; Schoch, K.; Goldstein, D.B.; Howard, T.D.; Berry, M.N.; Schwartz, C.E.; Cronin, K.; Sliwa, S.; Allen, A.; et al. The RBMX gene as a candidate for the Shashi X-linked intellectual disability syndrome. *Clin. Genet.* **2015**, *88*, 386–390.

87. Corbett, M.A.; Dudding-Byth, T.; Crock, P.A.; Botta, E.; Christie, L.M.; Nardo, T.; Caligiuri, G.; Hobson, L.; Boyle, J.; Mansour, A.; et al. A novel X-linked trichothiodystrophy associated with a nonsense mutation in RNF113A. *J. Med. Genet.* **2015**, *52*, 269–274.

88. Klauck, S.M.; Felder, B.; Kolb-Kokocinski, A.; Schuster, C.; Chiocchetti, A.; Schupp, I.; Wellenreuther, R.; Schmötzer, G.; Poustka, F.; Breitenbach-Koller, L.; et al. Mutations in the ribosomal protein gene RPL10 suggest a novel modulating disease mechanism for autism. *Mol. Psychiatry* **2006**, *11*, 1073–1084.

89. Thevenon, J.; Michot, C.; Bole, C.; Nitschke, P.; Nizon, M.; Faivre, L.; Munnich, A.; Lyonnet, S.; Bonnefont, J.P.; Portes, V. Des; et al. RPL10 mutation segregating in a family with X-linked syndromic Intellectual Disability. *Am. J. Med. Genet. Part A* **2015**, *167*, 1908–1912.

90. Merienne, K.; Jacquot, S.; Pannetier, S.; Zeniou, M.; Bankier, A.; Gecz, J.; Mandel, J.L.; Mulley, J.; Sassone-Corsi, P.; Hanauer, A. A missense mutation in RPS6KA3 (RSK2) responsible for non-specific mental retardation [1]. *Nat. Genet.* **1999**, *22*, 13–14.

91. Dumitrescu, A.M.; Liao, X.-H.; Best, T.B.; Brockmann, K.; Refetoff, S. A Novel Syndrome Combining Thyroid and Neurological Abnormalities Is Associated with Mutations in a Monocarboxylate Transporter Gene (American Journal of Human Genetics (January 2004) 74 (168-175)). *Am. J. Hum. Genet.* **2004**, *74*, 168–175.

92. Friesema, E.C.H.; Grueters, P.A.; Biebermann, H.; Krude, H.; Von Moers, A.; Reeser, M.; Barrett, T.G.; Mancilla, E.E.; Svensson, J.; Kester, M.H.A.; et al. Association between mutations in a thyroid hormone transporter and severe X-linked psychomotor retardation. *Lancet* **2004**, *364*, 1435–1437.

93. Salomons, G.S.; Van Dooren, S.J.M.; Verhoeven, N.M.; Cecil, K.M.; Ball, W.S.; Degrauw, T.J.; Jakobs, C. X-linked creatine-transporter gene (SLC6A8) defect: A new creatine-deficiency syndrome. *Am. J. Hum. Genet.* **2001**, *68*, 1497–1500.

94. Masurel-Paulet, A.; Piton, A.; Chancenotte, S.; Redin, C.; Thauvin-Robinet, C.; Henrenger, Y.; Minot, D.; Creppy, A.; Ruffier-Bourdet, M.; Thevenon, J.; et al. A new family with an SLC9A6 mutation expanding the phenotypic spectrum of Christianson syndrome. *Am. J. Med. Genet. Part A* **2016**, *170*, 2103–2110.

95. Karaca, E.; Harel, T.; Pehlivan, D.; Jhangiani, S.N.; Gambin, T.; Coban Akdemir, Z.; Gonzaga-Jauregui, C.; Erdin, S.; Bayram, Y.; Campbell, I.M.; et al. Genes that Affect Brain Structure and Function Identified by Rare Variant Analyses of Mendelian Neurologic Disease. *Neuron* **2015**, *88*, 499–513.

96. Deardorff, M.A.; Kaur, M.; Yaeger, D.; Rampuria, A.; Korolev, S.; Pie, J.; Gil-Rodríguez, C.; Arnedo, M.; Loeys, B.; Kline, A.D.; et al. Mutations in cohesin complex members SMC3 and SMC1A cause a mild variant of Cornelia de Lange syndrome with predominant mental retardation. *Am. J. Hum. Genet.* **2007**, *80*, 485–494.

97. Zhang, Z.; Norris, J.; Kalscheuer, V.; Wood, T.; Wang, L.; Schwartz, C.; Alexov, E.; Van Esch, H. A Y328C missense mutation in spermine synthase causes a mild form of snyder-robinson syndrome. *Hum. Mol. Genet.* **2013**, *22*, 3789–3797.

98. Roll, P.; Rudolf, G.; Pereira, S.; Royer, B.; Scheffer, I.E.; Massacrier, A.; Valenti, M.P.; Roeckel-Trevisiol, N.; Jamali, S.; Beclin, C.; et al. SRPX2 mutations in disorders of language cortex and cognition. *Hum. Mol. Genet.* **2006**, *15*, 1195–1207.

99. Fassio, A.; Patry, L.; Congia, S.; Onofri, F.; Piton, A.; Gauthier, J.; Pozzi, D.; Messa, M.; Defranchi, E.; Fadda, M.; et al. SYN1 loss-of-function mutations in autism and partial epilepsy cause impaired synaptic function. *Hum. Mol. Genet.* **2011**, *20*, 2297–2307.

100. Tarpey, P.S.; Smith, R.; Pleasance, E.; Whibley, A.; Edkins, S.; Hardy, C.; O'Meara, S.; Latimer, C.; Dicks, E.; Menzies, A.; et al. A systematic, large-scale resequencing screen of X-chromosome coding exons in mental retardation. *Nat Genet* **2009**, *41*, 535–543.

101. O'Rawe, J.A.; Wu, Y.; Dörfel, M.J.; Rope, A.F.; Au, P.Y.B.; Parboosingh, J.S.; Moon, S.; Kousi, M.; Kosma, K.; Smith, C.S.; et al. TAF1 Variants Are Associated with Dysmorphic Features, Intellectual Disability, and Neurological Manifestations. *Am. J. Hum. Genet.* **2015**, *97*, 922–932.

102. Zemni, R.; Bienvenu, T.; Vinet, M.C.; Sefiani, A.; Carrié, A.; Billuart, P.; McDonell, N.; Couvert, P.; Francis, F.; Chafey, P.; et al. A new gene involved in X-linked mental retardation identified by analysis of an X;2 balanced translocation. *Nat. Genet.* **2000**, *24*, 167–170.

103. Nascimento, R.M.P.; Otto, P.A.; De Brouwer, A.P.M.; Vianna-Morgante, A.M. UBE2A, which encodes a ubiquitin-conjugating enzyme, is mutated in a novel X-linked mental retardation syndrome. *Am. J. Hum. Genet.* **2006**, *79*, 549–555.

104. Tarpey, P.S.; Raymond, F.L.; Nguyen, L.S.; Rodriguez, J.; Hackett, A.; Vandeleur, L.; Smith, R.; Shoubridge, C.; Edkins, S.; Stevens, C.; et al. Mutations in UPF3B, a member of the nonsense-mediated mRNA decay complex, cause syndromic and nonsyndromic mental retardation. *Nat Genet* **2007**, *39*, 1127–1133.

105. Tejada, M.I.; Villate, O.; Ibarluzea, N.; Hoz, A.-B.D. la; Martínez-Bouzas, C.; Beristain, E.; Martínez, F.; Friez, M.J.; Sobrino, B.; Barros, F. Molecular and clinical characterization of a novel nonsense variant in exon 1 of the UPF3B gene found in a large Spanish Basque family (MRX82). *Front Genet* **2019**, *10*, 1074.

106. Homan, C.C.; Kumar, R.; Nguyen, L.S.; Haan, E.; Raymond, F.L.; Abidi, F.; Raynaud, M.; Schwartz, C.E.; Wood, S.A.; Gecz, J.; et al. Mutations in USP9X

are associated with x-linked intellectual disability and disrupt neuronal cell migration and growth. *Am. J. Hum. Genet.* **2014**, *94*, 470–478.

107. Hoffjan, S.; Ibisler, A.; Tschentscher, A.; Dekomien, G.; Bidinost, C.; Rosa, A.L. WDR45 mutations in Rett (-like) syndrome and developmental delay : Case report and an appraisal of the literature. *Mol. Cell. Probes* **2016**, *30*, 44–49.

108. Hirata, H.; Nanda, I.; Van Riesen, A.; McMichael, G.; Hu, H.; Hambrock, M.; Papon, M.A.; Fischer, U.; Marouillat, S.; Ding, C.; et al. ZC4H2 mutations are associated with arthrogryposis multiplex congenita and intellectual disability through impairment of central and peripheral synaptic plasticity. *Am. J. Hum. Genet.* **2013**, *92*, 681–695.

109. Mansouri, M.R.; Marklund, L.; Gustavsson, P.; Davey, E.; Carlsson, B.; Larsson, C.; White, I.; Gustavson, K.H.; Dahl, N. Loss of ZDHHC15 expression in a woman with a balanced translocation t(X;15)(q13.3;cen) and severe mental retardation. *Eur. J. Hum. Genet.* **2005**, *13*, 970–977.

110. Moysés-Oliveira, M.; Guilherme, R.S.; Meloni, V.A.; Di Battista, A.; de Mello, C.B.; Bragagnolo, S.; Moretti-Ferreira, D.; Kosyakova, N.; Liehr, T.; Carvalheira, G.M.; et al. X-linked intellectual disability related genes disrupted by balanced X-autosome translocations. *Am. J. Med. Genet. Part B Neuropsychiatr. Genet.* **2015**, *168*, 669–677.

111. Masurel-paulet, A.; Kalscheuer, V.M.; Lebrun, N.; Hu, H.; Levy, F.; Chehadeh, S. El; Thauvin-robinet, C.; Thevenon, J.; Chancenotte, S.; Ruffier-bourdet, M.; et al. Expanding the Clinical Phenotype of Patients With a ZDHHC9 Mutation. **2013**.
