# Supplementary material for: Targeted Next-Generation Sequencing in Patients with Suggestive X-Linked Intellectual Disability
Source: Genes (Basel). 2020 Jan 2;11(1):51. doi: 10.3390/genes11010051 (PMC7017351; doi:10.3390/genes11010051)
Supplement: Supplementary file 1 [file genes-11-00051-s001.zip › Genes-672756-Ibarluzea-Table_S2.pdf]

Table S2. Technical sequencing data.

	Bases	>Q20	Reads	Mean Read Length (bp)	Mapped Reads	On Target (%)	Mean read Depth	Uniformity (%)	1x	20x	100x
**ID1010**	172.336.456	150.381.667	707.609	243	706.532	97,53	243,60	94,11	99,01	96,55	87,29
**ID1011**	173.654.359	151.361.505	720.241	241	719.198	96,91	244,40	94,20	99,18	96,76	87,27
**ID1012**	182.762.521	160.227.812	745.785	245	744.697	96,47	256,40	94,65	99,13	96,79	89,55
**ID1013**	189.606.935	165.318.041	788.709	240	787.504	96,08	265,10	94,59	99,08	96,73	89,85
**ID1014**	182.873.265	160.110.795	751.080	243	749.791	96,60	256,90	94,67	99,14	96,68	89,20
**ID1015**	202.276.662	177.111.831	826.559	244	825.458	96,82	284,50	94,55	99,21	96,91	90,61
**ID1016**	190.270.717	16.258.545	787.297	241	786.004	96,16	266,40	94,43	99,13	96,75	89,37
**ID1121**	181.024.729	163.690.936	690.857	262	689.382	96,30	253,70	94,41	99,16	97,26	86,73
**ID1122**	196.671.250	178.399.923	743.869	264	742.496	96,17	275,40	94,88	99,15	97,42	89,61
**ID1123**	188.994.839	170.565.341	721.035	262	719.776	96,39	264,90	95,04	99,19	97,46	89,62
**ID1124**	183.474.581	165.691.233	699.832	262	698.591	96,29	257,00	95,06	99,32	97,46	88,86
**ID1125**	204.477.709	184.626.622	776.880	263	775.480	96,52	286,70	94,79	99,20	97,42	90,06
**ID1126**	200.027.452	180.238.028	767.504	260	765.898	96,71	281,50	94,15	99,20	97,27	87,89
**ID1127**	194.151.975	175.578.728	741.095	261	739.779	96,17	271,60	94,84	99,22	97,38	89,85
**ID1128**	226.380.173	203.784.703	852.605	265	851.508	96,79	318,80	94,75	99,21	97,54	91,80
**ID1129**	138.782.591	125.493.142	528.309	262	527.220	96,55	195,00	94,79	99,19	96,77	81,36
**ID1210**	212.969.472	188.801.480	834.085	255	832.110	96,21	298,20	94,25	99,22	97,44	89,32
**ID1203**	153.959.274	137.398.085	592.561	259	591.498	95,42	214,10	94,99	99,09	96,96	84,20
**ID1204**	187.145.229	166.149.146	734.483	254	732.916	95,63	260,70	95,08	99,31	97,32	89,14
**ID1205**	192.693.038	170.668.010	756.428	254	754.897	95,38	268,40	95,13	99,22	97,44	89,93
**ID1206**	192.591.059	171.731.541	745.192	258	743.813	95,53	268,10	95,09	99,23	97,32	90,11
**ID1207**	172.091.117	153.045.049	674.996	254	673.552	95,56	239,80	94,86	99,19	97,14	87,88
**ID1208**	214.838.518	189.829.745	852.449	252	850.553	95,57	299,20	94,81	99,18	97,51	90,72
**ID1209**	222.570.770	197.170.231	875.273	257	873.533	95,91	310,50	94,30	99,23	97,49	89,94
**ID1301**	134.513.017	121.686.559	538.597	249	537.426	95,67	188,00	93,00	99,08	95,64	76,27
**ID1302**	181.004.063	165.026.183	688.960	262	687.605	95,61	252,70	95,00	99,25	97,40	87,71
**ID1303**	164.147.174	150.243.280	611.968	268	611.026	96,00	229,60	95,09	99,17	97,28	86,48

**ID1304**	200.431.561	182.589.436	750.351	267	749.293	95,94	280,10	95,10	99,31	97,67	90,31
**ID1305**	203.873.194	185.310.210	775.110	263	773.839	95,02	282,80	95,25	99,41	97,76	91,47
**ID1306**	213.353.669	195.139.902	794.718	268	793.470	95,55	297,60	95,34	99,35	97,78	91,63
**ID1307**	207.092.403	189.183.619	774.531	267	773.182	96,12	289,90	94,56	99,23	97,48	89,31
**ID1308**	238.768.282	216.804.170	907.496	263	905.598	95,98	334,50	94,93	99,33	97,85	92,30
**ID1309**	151.599.911	138.158.919	573.465	264	572.330	95,60	211,70	95,13	99,25	97,23	84,47
**ID1401**	203.840.979	182.807.972	764.907	266	764.212	97,03	287,60	93,52	99,18	97,43	87,14
**ID1402**	197.007.741	177.222.986	740.589	266	739.717	96,44	276,50	94,40	99,28	97,29	88,10
**ID1403**	188.534.597	170.391.797	707.718	266	706.819	96,48	254,90	94,54	99,25	97,31	88,11
**ID1404**	245.362.497	220.512.343	922.456	265	921.413	96,32	344,40	94,73	99,26	97,71	92,30
**ID1405**	255.159.169	229.055.358	964.750	264	963.625	96,45	358,60	94,46	99,32	97,74	92,25
**ID1406**	232.202.677	209.908.493	864.299	268	863.443	96,32	326,00	94,04	99,31	97,51	90,60
**ID1407**	246.784.204	222.589.160	925.500	266	924.546	96,44	346,70	94,01	99,30	97,50	90,94
**ID0214**	215.496.766	185.704.605	1.002.191	215	996.809	95,89	301,60	91,66	98,87	95,35	86,07
**ID0216**	243.264.109	209.386.549	1.126.580	216	1.121.391	96,07	340,40	91,20	98,76	95,54	87,47
**ID0318**	177.975.033	154.813.416	772.924	230	770.401	94,49	246,70	93,52	98,99	96,13	85,73
**ID0319**	216.366.107	188.673.741	934.264	232	931.338	96,96	305,60	93,57	99,11	96,59	88,54
**ID0320**	236.867.002	205.674.956	1.027.427	231	1.024.562	97,51	336,20	92,96	99,04	96,53	89,10
**ID0321**	192.787.153	168.382.244	829.280	232	826.301	97,63	274,10	92,02	99,06	96,14	85,08
**ID0517**	174.896.087	151.037.659	784.295	223	779.641	95,94	246,30	92,32	99,04	96,02	83,64
**ID0606**	150.904.964	126.651.801	727.143	208	723.138	95,47	211,90	92,13	98,93	95,37	79,82
**ID0706**	230.802.725	212.713.526	859.860	268	859.037	97,12	326,70	94,48	99,11	97,01	92,13
**ID0707**	241.283.155	222.702.886	903.017	267	902.121	97,67	342,80	93,91	99,15	97,03	91,07
**ID0810**	167.874.019	151.182.915	635.360	264	634.407	96,64	236,20	93,43	98,95	96,45	85,63
**ID0811**	169.365.936	152.456.671	645.692	262	644.685	96,71	238,50	93,93	99,13	96,65	87,61
**ID0812**	168.529.620	152.704.110	636.772	264	635.951	96,05	236,00	94,61	99,12	96,62	88,21
**ID0813**	211.898.868	190.966.206	807.244	262	805.862	96,19	297,50	93,78	99,17	96,71	89,24
**ID0814**	201.872.893	226.380.173	763.368	264	762.228	95,63	281,90	93,52	99,02	96,69	88,54
**ID0808**	162.661.167	145.968.914	623.171	261	622.209	96,59	229,00	93,68	99,16	96,31	86,35
**ID0919**	222.648.667	198.859.731	859.060	259	857.811	97,22	314,40	93,72	99,18	97,09	89,98
**ID0921**	250.040.631	224.325.707	958.266	260	956.565	97,12	352,80	94,06	99,20	97,07	91,50
**ID0922**	291.221.340	261.731.357	1.102.581	264	1.100.866	97,06	411,10	94,43	99,09	97,37	93,32
**ID0924**	214.538.756	192.045.948	823.137	260	822.020	97,34	303,40	94,17	99,18	96,88	90,40

**ID0925**	279.242.559	250.388.307	1.069.056	261	1.067.239	96,91	393,50	94,19	99,19	97,10	93,00
**MEAN**	200.669.465	177.590.393	788.702	255	787.120	96,31	281,46	94,21	99,17	97,00	88,56
**MIN**	134.513.017	16.258.545	528.309	208	527.220	94,49	188,00	91,20	98,76	95,35	76,27
**MAX**	291.221.340	261.731.357	1.126.580	268	1.121.391	97,67	411,10	95,34	99,41	97,85	93,32
